# Vascularized hiPSC-derived 3D cardiac microtissue on chip

**DOI:** 10.1016/j.stemcr.2023.06.001

**Published:** 2023-06-29

**Authors:** Ulgu Arslan, Marcella Brescia, Viviana Meraviglia, Dennis M. Nahon, Ruben W.J. van Helden, Jeroen M. Stein, Francijna E. van den Hil, Berend J. van Meer, Marc Vila Cuenca, Christine L. Mummery, Valeria V. Orlova

**Affiliations:** 1 Department of Anatomy and Embryology, Leiden University Medical Centre, 2333ZC Leiden, the Netherlands; 2 Department of Clinical Genetics, Leiden University Medical Center, 2333ZA Leiden, the Netherlands

**Keywords:** human induced pluripotent stem cells, hiPSCs, hiPSC-derived cardiomyocytes, cardiac microtissue, vascularization, cell-cell interaction, Organ-on-chip, Heart-on-chip

## Abstract

Functional vasculature is essential for delivering nutrients, oxygen, and cells to the heart and removing waste products. Here, we developed an *in vitro* vascularized human cardiac microtissue (MT) model based on human induced pluripotent stem cells (hiPSCs) in a microfluidic organ-on-chip by coculturing hiPSC-derived, pre-vascularized, cardiac MTs with vascular cells within a fibrin hydrogel. We showed that vascular networks spontaneously formed in and around these MTs and were lumenized and interconnected through anastomosis. Anastomosis was fluid flow dependent: continuous perfusion increased vessel density and thus enhanced the formation of the hybrid vessels. Vascularization further improved endothelial cell (EC)-cardiomyocyte communication via EC-derived paracrine factors, such as nitric oxide, and resulted in an enhanced inflammatory response. The platform sets the stage for studies on how organ-specific EC barriers respond to drugs or inflammatory stimuli.

## Highlights

• HiPSC-derived vascularized and perfusable cardiac MTs on-chip
• Fluid flow-dependent anastomosis of internal and external microvasculature
• EC-CM crosstalk via EC-derived paracrine factors

## Graphical abstract

**Figure:**
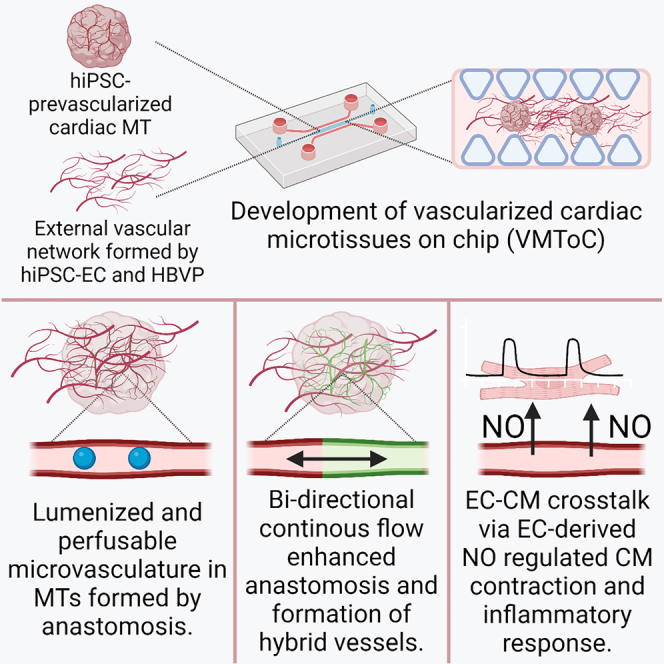

## Teaser

Arslan and colleagues developed vascularized and perfusable cardiac MTs on a microfluidic organ-on-chip platform (VMToC). The authors demonstrated that cardiac MTs can be efficiently vascularized within days, forming lumenized and perfusable vascular networks. Vascularization improved EC-CM communication via EC-paracrine factors and enhanced inflammatory responses. VMToC model provides a foundation for organ-specific cellular communication, drug screening, and disease modeling studies.

## Introduction

Cardiovascular disorders are a major cause of death around the world. The heart is a highly metabolic organ and has high energy demands for proper function. Endothelium is crucial in this process as it forms a semi-permeable barrier between cardiomyocytes (CMs) and the blood, providing selective nutrient, oxygen, and drug delivery to heart cells. It also mediates immune cell trafficking, for example, in the case of inflammation (Amersfoort et al., 2022). Interruption of the blood supply can lead to CM death, as in myocardial infarction (Berry and Duncker, 2020), and successful heart transplantation requires rapid restoration of blood supply to the donor heart.

Several 3D microphysiological models of the human heart have been described in which cell-cell crosstalk improved human pluripotent stem cell derived CM (hPSC-CM) maturation. These systems have proven useful for modeling some types of cardiac disease and for drug screening (reviewed in Arslan et al., 2022). Some of these models incorporated vascular networks inside engineered tissues, but in general, they were not perfusable by fluid or blood (equivalents) (Zhang et al., 2021). This has in part been addressed by either integrating organoids into a microfluidic chip or implanting them into living animals, as perfusion is also necessary for the stability and integrity of the microvascular networks (Mansour et al., 2018; Ryan et al., 2021; Takebe et al., 2013).

In the study here, we developed a fully vascularized and perfusable cardiac microtissue (MT) on-chip by integrating pre-vascularized cardiac MTs with an external vascular network formed by self-organization of human induced pluripotent stem cell-derived endothelial cells (hiPSC-ECs) and human brain vascular pericytes (HBVPs) in fibrin hydrogel in a microfluidic organ-on-chip. The integration of MTs in the chips did not adversely affect formation of the external, self-organized vascular network, and these networks developed robustly inside and around the MTs. Using this model, we demonstrated that cardiac MTs can be efficiently vascularized within days, and the lumenized vascular networks in and around the MTs can be perfused. This may ultimately allow selective nutrient or drug delivery to the cells via the endothelial barrier inside the MTs, mimicking heart tissue *in vivo* even more closely. Notably, we demonstrated that EC-CM crosstalk can be modeled in vascularized cardiac MT on chip (VMToC) cultures. By using either L-N-nitro arginine methyl ester (L-NAME), an inhibitor of nitric oxide synthetases (NOSs), or pro-inflammatory stimulus, such as interleukin-1β (IL-1β), we revealed relevant paracrine signals playing roles in the regulation of cardiac function in MTs.

## Results

### Characterization of the vascular network in the presence of cardiac MTs

**Figure 1:**
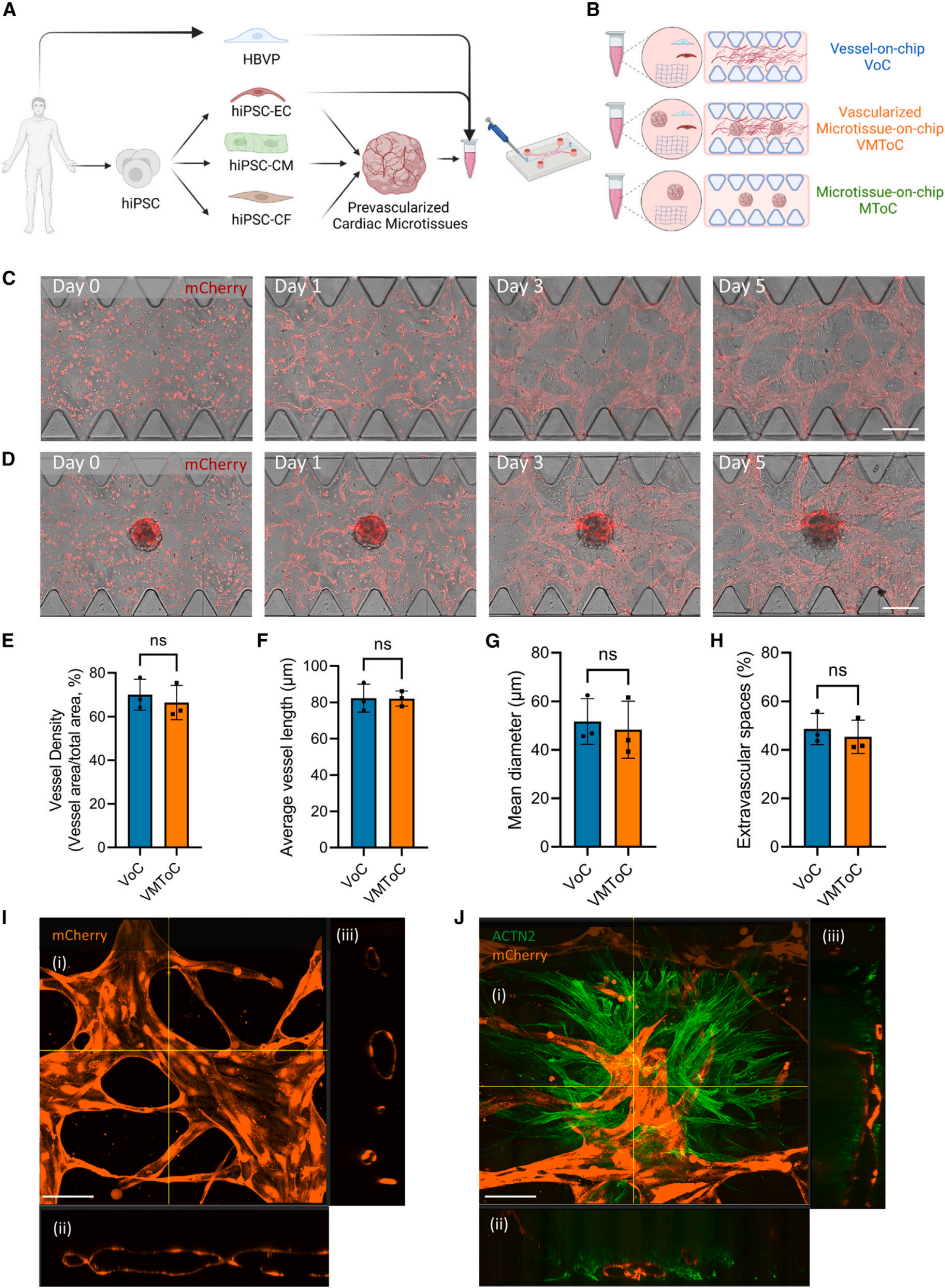
Characterization of the vascular network in the presence of cardiac MTs (A and B) Schematic overview of the vascularized cardiac MTs experimental setup (A) and conditions used in the study (B). hiPSC-ECs and HBVPs were cocultured to form VoC; hiPSC-MTs were cocultured with hiPSC-ECs and HBVPs to form VMToC; hiPSC-MTs were integrated into chips without any additional vascular cells to form MToC. (C and D) Representative images from chips on days 0, 1, 3, and 5 after seeding in the chips, which shows the development of external vascular networks in VoC (C) and VMToC (D). Images showing bright field and hiPSC-EC (red, mCherry) (10×). Scale bars, 300 μm. (E–H) Quantification of vessel density (%) (E), average vessel length (μm) (F), mean diameter (μm) (G), extravascular spaces (%) (H). Error bars are shown as mean ± SD from N = 3; three independent experiments with at least six microfluidic channels per experiment. Student’s t test; ns, not significant. (I and J) Representative confocal images of microvascular network in VoC (I) and VMToC (J) showing hiPSC-ECs (orange, mCherry) and hiPSC-CMs (green, ACTN2). Images displaying maximum projection in xyz (i), xy (ii), and yz cross-sectional perspectives (iii) (40×). Scale bar, 100 μm. See also Figure S1 and Videos S1, S2, and S3.

The VMToC model was generated by combining pre-vascularized cardiac MTs (Giacomelli et al., 2020; Campostrini et al., 2021) with hiPSC-ECs and HBVPs in a fibrin hydrogel. This MT/cell/hydrogel mix was then embedded in the gel channel of commercially available AIM Biotech 3D cell culture chips (Figure 1A). To investigate the effect of cardiac MTs on vascular network formation, we compared the VMToC model with a standard vessel-on-chip (VoC) that included hiPSC-EC and HBVP cocultures but without cardiac MTs (Figure 1B). Vascular organization in VoCs and VMToCs was already evident on day 1 (Figures 1C and 1D). On day 3, a complex vascular network had formed in the chips, and these networks were stabilized by day 5. Quantification of the vascular network parameters such as vessel density (Figure 1E), average vessel length (Figure 1F), mean vessel diameter (Figure 1G), and extravascular space (Figure 1H) showed no significant differences between VoC and VMToC conditions. Vascular networks in VoC (Figure 1I) and the microvasculature in MTs in VMToC (Figure 1J, Video S1) were both lumenized. To investigate whether the lumenized vasculature in cardiac MTs was actually perfusable, fluorescent beads (2 μm) were introduced into the medium in one of the media inlets of the chips. Beads entered the vascular networks from the intersection of medium-gel channel and moved spontaneously to the microvasculature in MTs (Figure S1A). Beads stayed within the microvasculature contours in the MTs indicating the interconnected vessels in the MTs were indeed perfusable (Figures S1B–S1E, Videos S2 and S3).

Video S1. 3D reconstruction of VMToC on the left showing lumenized microvascular network formed by hiPSC-ECs (orange, mCherry) embedded in hiPSC-CMs (green, ACTN2) 3D reconstruction of MToC on the right showing that hiPSC-ECs (orange, mCherry) migrated out of the MTs, and hiPSC-CMs (green, ACTN2) developed long protrusions, related to Figures 1 and 3.

Video S2. Simultaneous video recording of two channels for contraction (on the left) and perfusion of fluorescent beads (on the right) showing hiPSC-CMs (green, ACTN2) and hiPSC-ECs (orange, mCherry) (20×), related to Figure 1

Video S3. Simultaneous video recording of two channels for contraction (on the left) and perfusion of fluorescent beads (on the right) showing hiPSC-CMs (green, ACTN2) and hiPSC-ECs (orange, mCherry) (40×), related to Figure 1

### Mechanism of the intra-microvascular network formation

**Figure 2:**
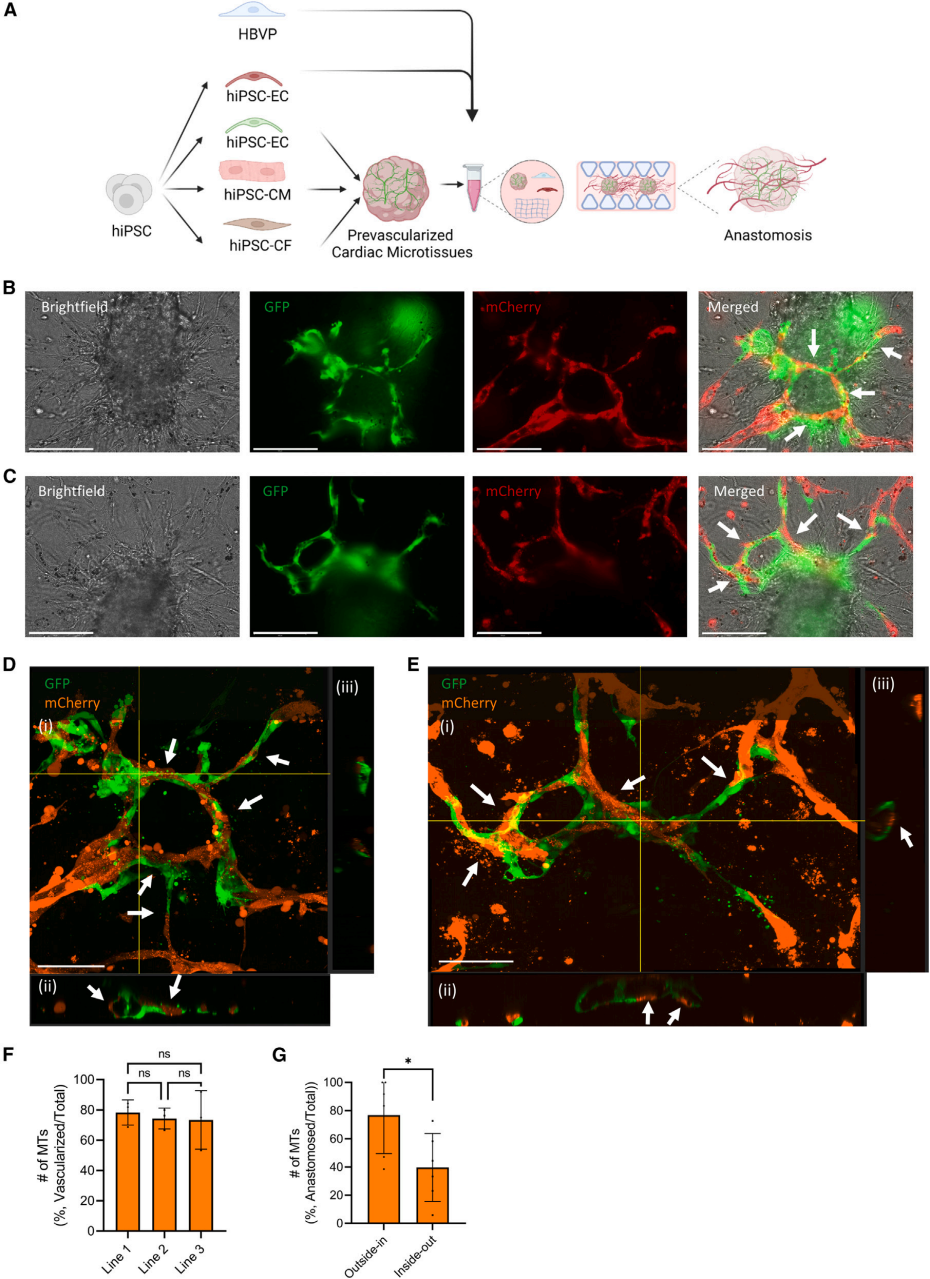
Mechanism of the intra-microvascular network formation (A) Schematic of the experimental setup. MTs were generated combining hiPSC-CMs, hiPSC-ECs (green, GFP), and hiPSC-CFs. These MTs were cocultured with hiPSC-ECs (red, mCherry) and HBVPs in chips. (B and C) Representative images of outside-in (B) and inside-out (C) anastomosis and formation of hybrid vessels by interconnection of internal microvascular network (green, GFP) and external vascular network (red, mCherry) (20×). Scale bar, 150 μm. (D and E) Representative confocal images of hybrid vessels visible in (B) and (C), respectively. Internal hiPSC-ECs (green, GFP) and external hiPSC-ECs (orange, mCherry). Images displaying maximum projection in xyz (i), xy (ii), and yz cross-sectional perspectives (iii) (40×). Scale bar, 100 μm. Arrows indicate the anastomosed points and hybrid lumens. (F) Quantification of number of vascularized MTs (%, number vascularized MTs/total number of MTs). MTs contained CMs from three different hiPSC lines. Error bars are shown as mean ± SD from N = 3; three independent experiments with at least 10 MTs in each experiment. One-way ANOVA; ns, not significant. (G) Quantification of number of anastomosed MTs (%, #anastomosed MTs/total # of MTs) in independent experiments. Error bars are shown as mean ± SD from N = 6; six independent experiments with at least nine MTs in each experiment. Student’s t test, ∗p < 0.05. See also Figure S2.

In order to investigate the mechanism of the intra-microvascular network formation, we used hiPSC-ECs derived from two different fluorescent reporter lines (expressing mCherry or GFP). GFP-expressing hiPSC-ECs were used to prevascularize the cardiac MTs, and these MTs were cocultured with mCherry-expressing hiPSC-ECs and HBVPs in the chips (Figure 2A). Internal microvasculature in the MTs and external vascular network around MTs underwent anastomosis as early as day 2. Anastomosis was bidirectional in the system: (1) outside-in anastomosis where the external network invaded the MTs and anastomosed with the internal microvasculature in the MTs (Figures 2B and 2D) and (2) inside-out anastomosis where part of the internal microvascular network migrated outside the MT and anastomosed with the external vascular network (Figures 2C and 2E). As a result, hybrid vessels composed of hiPSC-ECs partly from MTs and partly from external vascular networks were formed. Confocal images showed that these hybrid vessels were lumenized (Figures 2D and 2E). The number of vascularized tissues was comparable in all independent experiments (Figure 2F), with higher numbers of MTs showing evidence of outside-in anastomosis than those showing inside-out anastomosis (Figure 2G).

Since fluid flow is known to promote EC migration, proliferation, and survival (Abe et al., 2019; Galie et al., 2014; Zhang et al., 2022), we tested whether we could enhance the vascularization of cardiac MTs by introducing perfusion using a rocker (Figure S2A). Both intermittent and continuous perfusion resulted in anastomosis between the internal microvasculature and external vascular networks (Figures S2B and S2C, respectively). However, continuous perfusion resulted in higher mCherry+ and GFP+ vessel density (Figures S2D and S2E) in the chips. Intermittent perfusion resulted in the formation of hybrid vessels where only a minority of cells originated in the cardiac MTs (Figure S2F). This improved substantially under continuous perfusion (Figure S2G).

### Characterization of cardiac MTs in the absence or presence of external vascular network

**Figure 3:**
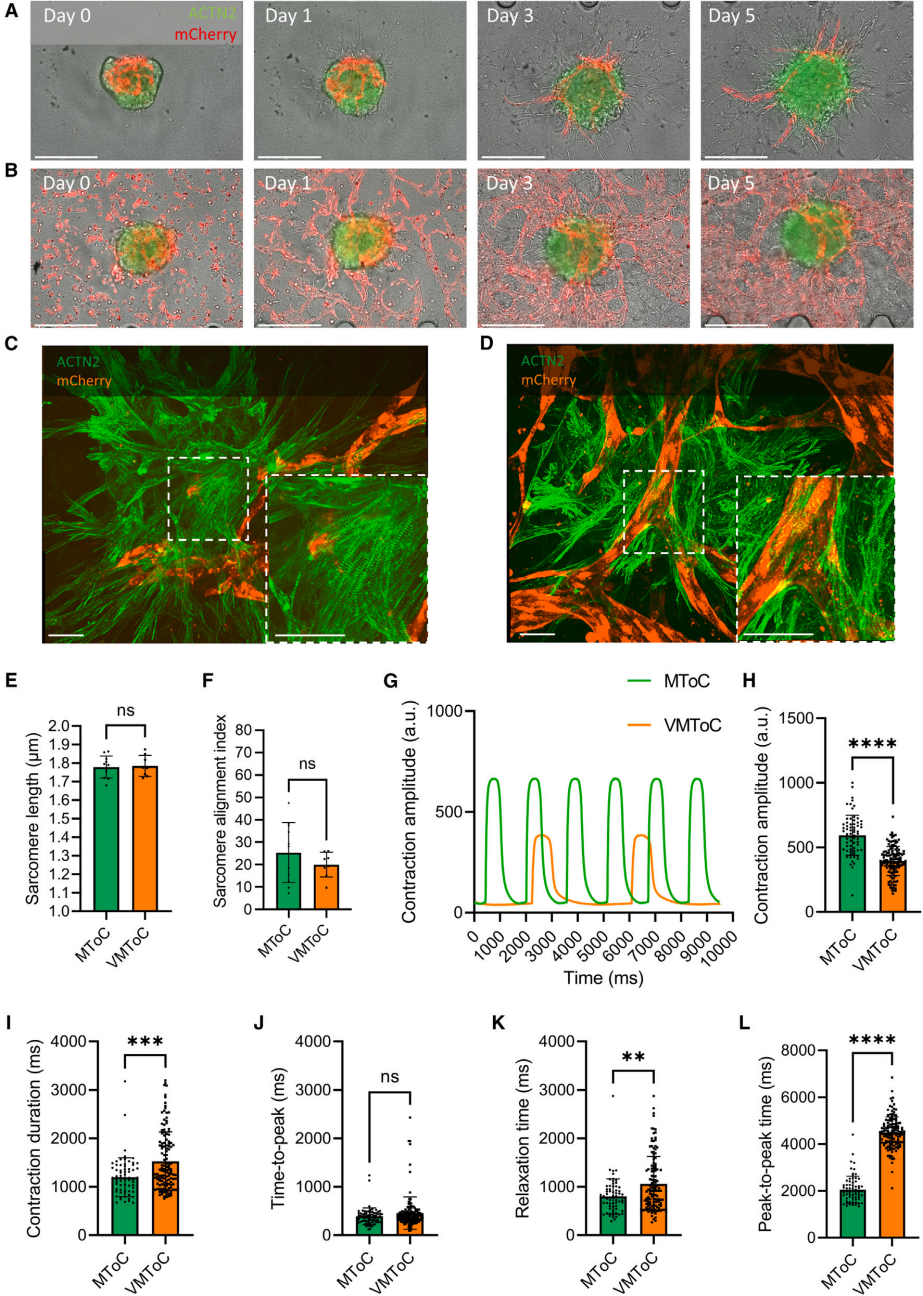
Characterization of contractile dynamics of cardiac MTs in MToC and VMToC (A and B) Representative images of MTs in chips on days 0, 1, 3, and 5 in MToC (A) and VMToC (B) (10×). Scale bar, 300 μm. (C and D) Representative confocal images of sarcomeres showing hiPSC-ECs (orange, mCherry) and hiPSC-CMs (green, ACTN2) in MToC (C) and VMToC (D) (40×). White dashed box is the area that is zoomed 100×. Scale bar, 50 μm. (E and F) Quantification of the sarcomere parameters: sarcomere length (E); sarcomere alignment index (F); error bars are shown as mean ± SD from MToC N = 3, n = 11; VMToC N = 4, n = 8; three or four independent experiments with at least eight MTs. (G) Representative beating traces of MTs from MToC (green trace) and VMToC (orange trace). (H–L) Quantification of the contraction parameters: contraction amplitude (H), contraction duration (I), time to peak (J), relaxation time (K), and peak-to-peak time (L) in MTs with AICS-0075 hiPSC-CMs. Error bars are shown as mean ± SD from MToC N = 3, n = 69; VMToC N = 3, n = 132; three independent experiments with 16 or 24 MTs from at least six different microfluidic channels each experiment. Student’s t test (E), Wilcoxon-Mann-Whitney test (F and H–L). ∗∗∗p < 0.001, ∗∗∗∗p < 0.0001; ns, not significant. See also Figure S3.

We next examined cardiac MTs in the chips for any changes in their sarcomere organization and contractile properties. MTs were compared in the absence (MToC, Video S1, Figures 1B and 3A) or presence (VMToC, Figure 3B) of an external vascular network. Sarcomere morphologies appeared similar in the MToC (Figure 3C) and VMToC (Figure 3D), which was confirmed by similar sarcomere lengths (Figure 3E) and alignment indices (Figure 3F) assessed using the SOTATool (Stein et al., 2022). This indicated that there was no additional effect on sarcomere organization in the presence of external vascular network.

Contractility of cardiac MTs was analyzed using the video-based software tool MUSCLEMOTION (Sala et al., 2018). Representative beating traces showed that MTs under both conditions maintained their contractility in hydrogel (Figure 3G). However, contraction amplitude (Figure 3H) was significantly lower in VMToC, and contraction duration (Figure 3I), relaxation time (Figure 3K) and peak-to-peak time (Figure 3L) were significantly longer in VMToC. Time-to-peak (Figure 3J) was similar in both conditions. The changes in these parameters were consistent in VMToC and MToC that were generated using a second, independent hiPSC line for CMs (Figures S3A–S3E). In order to test whether the increase in contraction duration and relaxation time in the VMToC is not rate dependent, we performed electrical pacing in the chips using custom-made electrodes that fit the gel channel inlet and outlet. When these MTs were paced at 0.8 Hz and 1 Hz (Figures S3F and S3G), contraction duration at 90% and 50% transient was significantly higher in VMToC compared with MToC, consistent with the results under conditions of spontaneous beating (Figures S3H and S3I). We further investigated if the duration differences could be explained by time-to-peak or relaxation time changes. However, these parameters were highly variable and were not significantly different between VMToC and MToC even when paced (Figures S3J and S3K).

### Modeling EC-CM crosstalk in cardiac MTs

**Figure 4:**
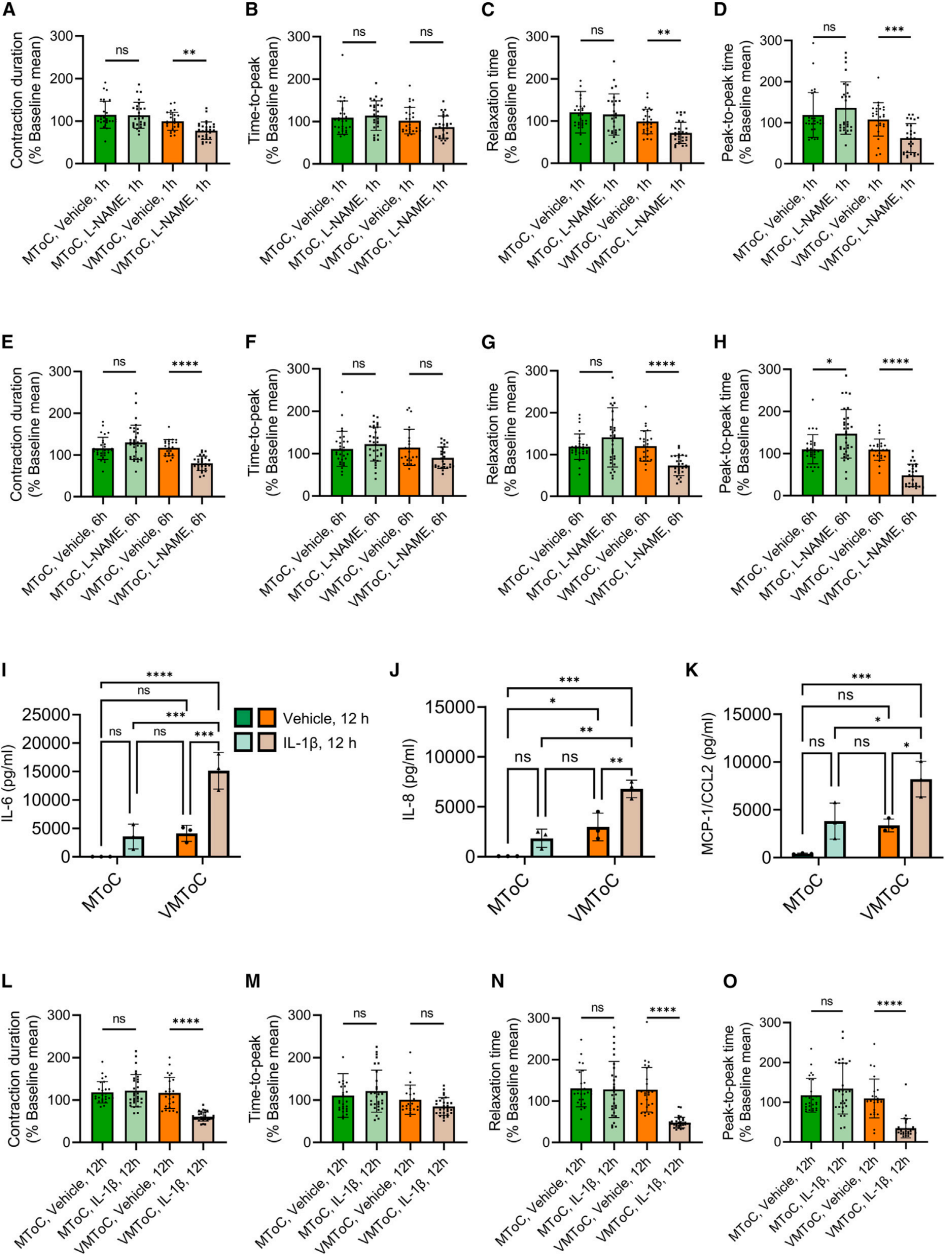
Altered contractile dynamics and inflammatory response of cardiac MTs regulated by EC-CM communication in VMToC but not in MToC (A–H) Quantification of the contraction parameters presented as percentage change from the baseline mean of spontaneous beating condition, after 1 h (A–D) and 6 h (E–H) incubation with vehicle or L-NAME (1 mM): contraction duration (A and E); time to peak (B and F); relaxation time (C and G); peak-to-peak time (D and H) in MTs with LUMC0059iCTRL03 hiPSC-CMs. Error bars are shown as mean ± SD from MToC N = 3, n > 28 (vehicle) and n > 27 (L-NAME); VMToC N = 3, n > 24 (vehicle) and n > 27 (L-NAME); three independent experiments with at least seven MTs in each experiment. (I–K) Quantification of pro-inflammatory cytokines from the medium after 12 h incubation of IL-1β (10 ng/mL): IL-6 (I); IL-8 (J); MCP1/CCL2 (K) in MTs with LUMC0059iCTRL03 hiPSC-CMs. Y axis shows concentration (pg/mL). Error bars are shown as mean ± SD. MToC N = 3, n > 3; VMToC N = 3, n > 3; three independent experiments; medium was collected from at least with three different microfluidic channels in each experiment. (L–O) Quantification of the contraction parameters presented as percentage change from the baseline mean of spontaneous beating condition after 12 h incubation with vehicle or IL-1β (10 ng/mL): contraction duration (L); time to peak (M); relaxation time (N); peak-to-peak time (O) in MTs with LUMC0059iCTRL03 hiPSC-CMs. Error bars are shown as mean ± SD from MToC N = 3, n = 26 (vehicle) and n = 30 (IL-1β); VMToC N = 3, n = 25 (vehicle) and n = 31 (IL-1β); three independent experiments with at least seven MTs in each experiment. Kruskal-Wallis test with Dunn’s multiple comparisons test (A–H, L–O), two-way ANOVA with Šidák’s multiple comparisons test (I–K), ∗p < 0.05, ∗∗p < 0.01, ∗∗∗p < 0.001; ∗∗∗∗p < 0.0001; ns, not significant. See also Figure S4.

To explore whether VMToC has added value over MToC in studying inflammation, we investigated whether EC-CM crosstalk can be modulated by either (1) L-NAME, a nonselective inhibitor of NOS, or (2) a pro-inflammatory stimulus, such as IL-1β. MToC and VMToC were incubated with L-NAME or vehicle for 1 h (Figures 4A–4D and S4A–S4D) or 6 h (Figures 4E–4H and S4E–S4H). L-NAME had no effect on the contraction time parameters: duration, time to peak, and relaxation time in MToC after 1- or 6-h incubation compared with the vehicle alone. By contrast, 6-h L-NAME exposure of VMToC cultures decreased contraction duration (Figures 4E and S4E) and relaxation time (Figures 4G and S4G) and had no effect on the time to peak (Figures 4F and S4F). 1 h of L-NAME incubation was more variable between the lines tested and decreased contraction duration and relaxation time in one but not the other control line (Figures 4A, 4C, S4A, and S4C). Peak-to-peak times appeared to decrease in one of the control lines tested after 1 and 6 h but not the other (Figures 4D, 4H, S4D, and S4H).

Finally, MToC and VMToC were stimulated for 12 h with IL-1β (10 ng/mL) to investigate the effect of pro-inflammatory cytokines. Pro-inflammatory cytokine release and contractile parameters were assessed. Stimulation with IL-1β resulted in significant upregulation of IL-6 (Figure 4I), IL-8 (Figure 4J), and cytokine chemoattractant protein 1 (MCP-1/CCL2, Figure 4K) in VMToC but not in MToC. In addition, IL-1β stimulation decreased contraction duration, relaxation time, and peak-to-peak time in VMToC but had no effect on MToC (Figures 4L–4O).

## Discussion

In the present study, we established vascularized and perfusable cardiac MT on a VMToC with vascular cells self-organized in fibrin hydrogel. The external vascular cells reproducibly formed a continuous vascular network in and around the MTs, and this was not affected by contraction of the MTs. Continuous perfusion generated by placing the chips on a rocker resulted in greater vascularization, improved anastomosis, and enhanced formation of hybrid vessels. Since mechanistic studies on molecular pathways mediating anastomosis are challenging *in vivo*, this platform could provide an alternative way to understand this process *in vitro*. Furthermore, these networks developed robust lumens that contracted rhythmically in synchrony with surrounding CMs. This rhythmic contraction of CMs resulted in the generation of bidirectional flow as indicated by oscillatory movement of fluorescent beads into and through the organized vascular network inside of the MT. Currently, no animal models are available that model ischemic heart disease caused by coronary microvascular obstruction (Niccoli et al., 2016; Sorop et al., 2020). Therefore, vascularized and perfusable cardiac MTs could be useful in studying mechanisms underlying microvascular obstruction using cardiac and vascular cells derived from patients.

Previously we demonstrated that including ECs and cardiac fibroblasts (CFs) in MTs enhances CM maturation (Giacomelli et al., 2020). However, no differences in sarcomere organization were found between VMToC and MToC, indicating that further enhancement was not induced by the external vascular network. VMToCs showed characteristic low beat rates, but nevertheless, they continued to contract rhythmically throughout the experiments. Analysis of VMToC contractile parameters showed increased contraction duration and relaxation times. Notably, the increase in contraction duration in VMToC was not rate dependent and was maintained upon pacing at 0.8 and 1 Hz.

We further demonstrated that EC-CM crosstalk could be captured in VMToC upon delivery of stimuli via the external vascular network. Inhibition of NOS using L-NAME in VMToCs reversed the slow beating and decreased contraction duration, relaxation, and peak-to-peak time. At the same time, L-NAME had no significant effect in MToC after 1- or 6-h incubation, except for an increase in peak-to-peak time. The effect of L-NAME in VMToC could be explained by a possible increase in endothelium-derived endothelin (ET-1) production upon NOS inhibition (Czóbel et al., 2009; Kourembanas et al., 1993). Since VMToCs contain more ECs than MToCs, this could result in a pronounced response to NOS inhibition that was not observed in MToCs. Similarly, stimulation with IL-1β for 12 h resulted in upregulation of several pro-inflammatory cytokines, such as IL-6, IL-8, and MCP-1 in VMToC, but not in MToC, suggesting that only the vascular units were effectively responding to pro-inflammatory cytokine stimulation that are known contributors to EC dysfunction and heart failure. Stimulation with IL-1β decreased contraction duration, relaxation time, and peak-to-peak time. This was somewhat unexpected since IL-1β and IL-6 are known as negative inotropes that increase NO levels via upregulating the expression of inducible NOS (iNOS) in isolated CMs (Segers et al., 2018). On the other hand, IL-1β and IL-6 decrease endothelial NOS (eNOS) expression and activity in ECs (Hung et al., 2010; Kawasaki et al., 2015; Saura et al., 2006). Although we have not examined expression of iNOS and eNOS upon IL-1β treatment in VMToC vs. MToC, the fact that the IL-1β inhibitory effect was only observed in VMToC indicates the importance of the vascular component in mediating the response in CMs.

Finally, although the formation of the vascular networks was robust in VMToC, we observed some batch-to-batch and plate-to-plate variability in the degree of vascularization. Vascularization of MTs was mostly through the anastomosis, so the extent of pre-vascularization in MTs might contribute to variability. In some cases where MTs were not well-vascularized, vascularization was improved by introducing continuous perfusion during later stages of culture (data not shown). In addition, inter-batch and inter-line variability might be the result of differences in basal contractile parameters and/or drug responses in VMToCs. We showed consistency between two different hiPSC lines, but further validation using more hiPSC lines may be of value as NOS levels may differ between CMs and ECs from different lines and thus affect contractile dynamics and drug responses to different extents.

In summary, we demonstrated that 3D cardiac MTs can be integrated into microfluidic chips with the external vascular network formed by hiPSC-ECs and HBVPs. Using this model, we demonstrated that cardiac MTs can be efficiently vascularized within days, and the lumenized vascular networks in and around the MTs can be perfused. This may ultimately allow selective nutrient or drug delivery to the cells via the endothelial barrier inside the MTs, mimicking heart tissue *in vivo* even more closely. Our vascularized cardiac MT model thus provides a foundation for studies on organ-specific cellular communication, specifically for the endothelial barrier, drug screening, and disease modeling.

## Experimental procedures

### Resource availability

#### Corresponding author

Requests for further information or more detailed protocols should be directed to and will be fulfilled by the corresponding author, Valeria V. Orlova (v.orlova@lumc.nl).

#### Materials availability

This study did not generate new unique reagents.

### hiPSC lines

The medical ethical committee in Leiden University, the Netherlands, approved the use of hiPSCs in this study. A detailed list of the hiPSC lines and batches used for each experiment can be found in Table S1.

### 3D cardiac microtissue formation

3D cardiac MTs were formed using hiPSC-CMs, hiPSC-ECs, and hiPSC-CFs following a previously established protocol (Giacomelli et al., 2020).

### Microfluidic chip culture

Cell preparation prior to chip seeding is described in the supplemental information. Commercially available microfluidic chips (AIM Biotech) were used. Cell and MT mixtures were prepared as follows: (1) four MTs/channel in combination with 25 × 10^6^ hiPSC-ECs/mL and 5 × 10^6^ HBVP cells/mL (5:1 ratio) (VMToC); (2) 25 × 10^6^ hiPSC-ECs/mL and 5 × 10^6^ HBVP cells/mL (VoC) or (3) four MTs/channel (MToC) were resuspended in endothelial growth medium-2 (EGM-2, Lonza) supplemented with thrombin (4 U/mL) and then gently mixed with fibrinogen (final concentration 3 mg/mL, Sigma) at 1:1 vol ratio. Cell/hydrogel mixture was quickly loaded into the middle gel-loading channel of the microfluidic chip. Chips were incubated at room temperature for 15 min. To support vascular network formation in the presence of cardiac MTs, we used a mixture of CM and EC growth medium (bovine serum albumin and essential lipids medium and EGM-2, 50:50), supplemented with vascular endothelial growth factor (50 ng/mL). The γ-secretase inhibitor DAPT (10 μM) was also added to the medium on day 1 for 24 h. Intermittent gravity-driven flow in the whole chamber was induced by creating hydrostatic pressure through the addition of 100 μL medium to the right media ports and 50 μL media to left media ports in the medium channel. Medium was refreshed daily. For the continuous perfusion experiments, microfluidic chips were placed on the interval rocker platform (Perfusion rocker, MIMETAS) set at a 5° inclination angle and an 8-min interval from day 0 onward. Chips were maintained until day 7 and characterized between days 5 and 7.

### Statistical analysis

Statistical analysis was performed using GraphPad Prism 9. Student’s t test or one-way or two-way ANOVA for paired or unpaired measurements was applied as appropriate to test for differences in means between groups/conditions. Kruskal-Wallis test and Wilcoxon-Mann-Whitney test were used when the normality assumption did not hold. Data are expressed and plotted as the mean ± SD as indicated in figure legend. Detailed statistics and exact p values are indicated in each figure legend. Statistical significance was defined as p <0.05.

## Author contributions

Conceptualization, V.V.O.; methodology, U.A. and V.V.O.; software, U.A., D.N., J.S., and B.v.M.; validation, U.A., M.B., and V.M.; formal analysis, U.A.; investigation, U.A., M.B., V.M., D.M.N., R.W.J.v.H., J.M.S., F.E.v.d.H., B.v.M., and M.V.C.; visualization, U.A.; resources, C.L.M. and V.V.O.; writing – original draft, U.A., M.B., C.L.M., and V.V.O.; writing – review & editing, U.A., C.L.M., and V.V.O.; supervision, C.L.M., and V.V.O.; project administration, V.V.O.; funding acquisition, C.L.M. and V.V.O.

## Data Availability

Data will be shared with the research community upon request. No code or standardized datasets were generated.

## Supplemental information

Document S1. Figures S1–S4 and Table S1

Document S2. Article plus supplemental information
