# Vascularized hiPSC-derived 3D cardiac microtissue on chip

**DOI:** 10.1016/j.stemcr.2023.08.012

**Published:** 2023-10-10

**Authors:** Ulgu Arslan, Marcella Brescia, Viviana Meraviglia, Dennis M. Nahon, Ruben W.J. van Helden, Jeroen M. Stein, Francijna E. van den Hil, Berend J. van Meer, Marc Vila Cuenca, Christine L. Mummery, Valeria V. Orlova

## Main text

(Stem Cell Reports *18*, 1394–1404; July 11, 2023)

The authors regret the omission of a reference by King et al. (2022) that shows a heart-on-chip model that is similar to theirs but uses a combination of hiPSC-cardiomyocytes and primary human heart stromal cells. The reference appeared in the originally submitted version of the manuscript but was deleted in error during revision. It has now been restored in the article online.

