# Supplementary material for: Vascularized hiPSC-derived 3D cardiac microtissue on chip
Source: Stem Cell Reports. 2023 Jun 29;18(7):1394–404. doi: 10.1016/j.stemcr.2023.06.001 (PMC10362508; doi:10.1016/j.stemcr.2023.06.001)
Supplement: Document S1. Figures S1–S4 and Table S1 [file mmc1.pdf]

Stem Cell Reports, Volume *18*

Supplemental Information

Vascularized hiPSC-derived 3D cardiac microtissue on chip

Ulgu Arslan, Marcella Brescia, Viviana Meraviglia, Dennis M. Nahon, Ruben W.J. van Helden, Jeroen M. Stein, Francijna E. van den Hil, Berend J. van Meer, Marc Vila Cuenca, Christine L. Mummery, and Valeria V. Orlova

Inventory of Supplemental Information

Supplemental Figures and Legends:

Figure S1. Cardiac MTs induce pulsative intra-microvascular flow. Related to Figure 1.
Figure S2. Continuous perfusion enhances vascularization of cardiac MTs. Related to Figure 2.
Figure S3. Characterization of contraction parameters upon pacing. Related to Figure 3.
Figure S4. Altered contractile dynamics of cardiac MTs upon inhibition of EC-CM communication in VMToC but not in MToC. Related to Figure 4.

Supplemental Tables:

Supplemental Table 1. List of hiPSC lines and cell batches used per experiment.

Supplemental Videos:

Supplemental Video 1. 3D reconstruction of VMToC on the left showing lumenized microvascular network formed by hiPSC-ECs (orange, mCherry) embedded in hiPSC-CMs (green, ACTN2). 3D reconstruction of MToC on the right showing that hiPSC-ECs (orange, mCherry) migrated out of the MTs and hiPSC-CMs (green, ACTN2) developed long protrusions. Related to Figure 1 and 3.

Supplemental Video 2. Simultaneous video recording of two channels for contraction (on the left) and perfusion of fluorescent beads (on the right) showing hiPSC-CMs (green, ACTN2) and hiPSC-ECs (orange, mCherry). (20x). Related to Figure 1.

Supplemental Video 3. Simultaneous video recording of two channels for contraction (on the left) and perfusion of fluorescent beads (on the right) showing hiPSC-CMs (green, ACTN2) and hiPSC-ECs (orange, mCherry). (40x). Related to Figure 1.

Supplemental Experimental Procedures

Supplemental References

Supplemental Figures and Legends

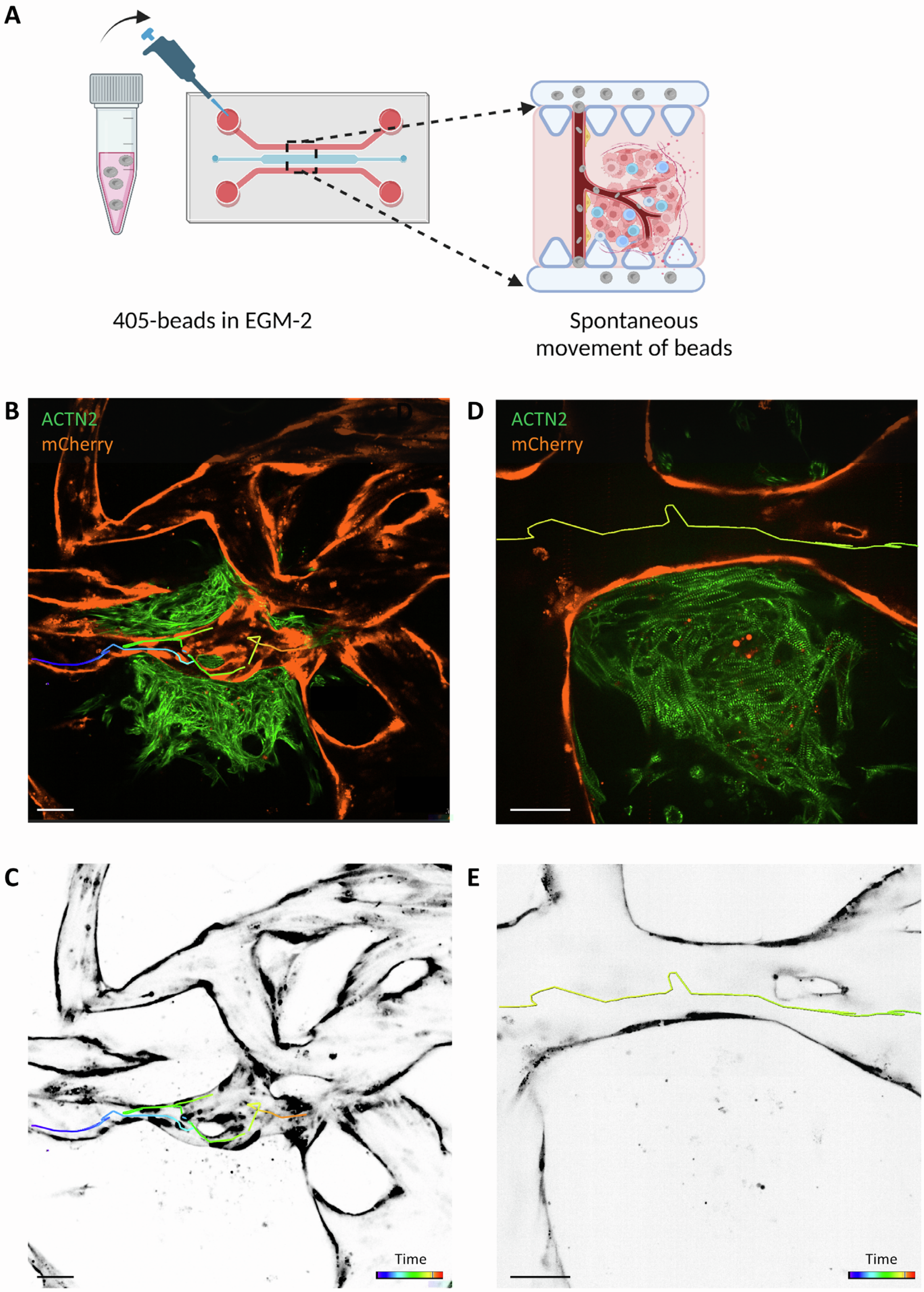

Figure S1. Cardiac MTs induce pulsative intra-microvascular flow. Related to Figure 1. (A) Schematic of the fluorescent beads (2 μm) perfusion experiment. Please see Supplemental Experimental Setup for experimental details. (B, D) Representative confocal images showing hiPSC-ECs (orange, mCherry) hiPSC-CM (green, ACTN2) and perfusion of 2 μm fluorescent beads (traces) in VMToC. (B is 20x and D is 40x with Scale bar, 50 μm. (C, E) Representative confocal images in black and white showing only vascular networks (black) and traces of beads inside (colored traces). Color coded bars represent time. Images are duplications of B and D. Scale bar, 50 μm.

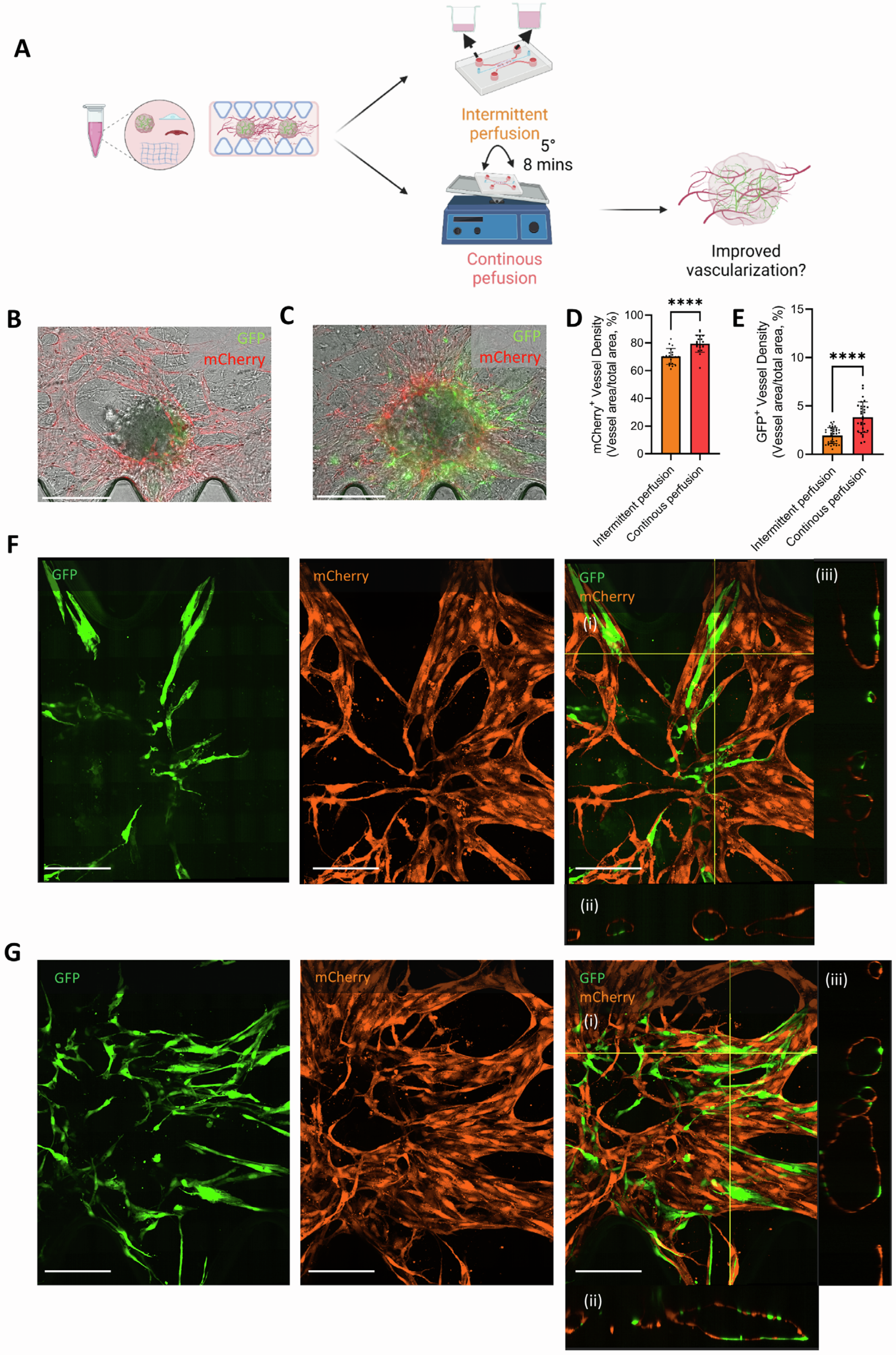
A
Intermittent perfusion
5°
8 mins
Continous pefusion
Improved vascularization?
B
GFP
mCherry
C
GFP
mCherry
D
mCherry+ Vessel Density (Vessel area/total area, %)
****
Intermittent perfusion
Continous perfusion
E
GFP+ Vessel Density (Vessel area/total area, %)
****
Intermittent perfusion
Continous perfusion
F
GFP
mCherry
(i)
(ii)
(iii)
G
GFP
mCherry
(i)
(ii)
(iii)

Figure S2. Continuous perfusion enhances vascularization of cardiac MTs. Related to Figure 2. (A) Schematic of the experimental setup for intermittent and continuous perfusion conditions. Chips were kept under intermittent gravity-driven flow with the medium exchange every 24 h or placed on the rocker (at a 5 degrees inclination angle and 8 min interval) to induce continuous perfusion through passive leveling between the reservoirs. (B, C) Representative images of VMToC under intermittent perfusion (B) and continuous perfusion (C) where anastomosis is visible by the formation of hybrid vessels (10x). Scale bar, 300 µm. (D) Quantification of $mCherry^+$ vessel density (%) in intermittent and continuous perfusion conditions. Error bars are shown as ±SD from N = 3, n = 19 (intermittent perfusion) and n = 20 (continuous perfusion); three independent experiments with at least six microfluidic channels per experiment. (E) Quantification of GFP^+ vessel density (%) in intermittent and continuous perfusion conditions. Error bars are shown as ±SD from N = 3, n = 35 (intermittent perfusion) and n = 29 (continuous perfusion); three independent experiments with at least six microfluidic channels per experiment. (F, G) Representative confocal images of hybrid vessels formed in intermittent perfusion (F) or continues perfusion (G) conditions. Internal hiPSC-EC (green, GFP), external hiPSC-EC (orange, mCherry). Images displaying xyz (i), xy (ii), and yz cross-sectional perspectives (iii) (40×). Scale bar, 150 µm.
Wilcoxon-Mann-Whitney test (D, E), ****$p < 0.0001$.

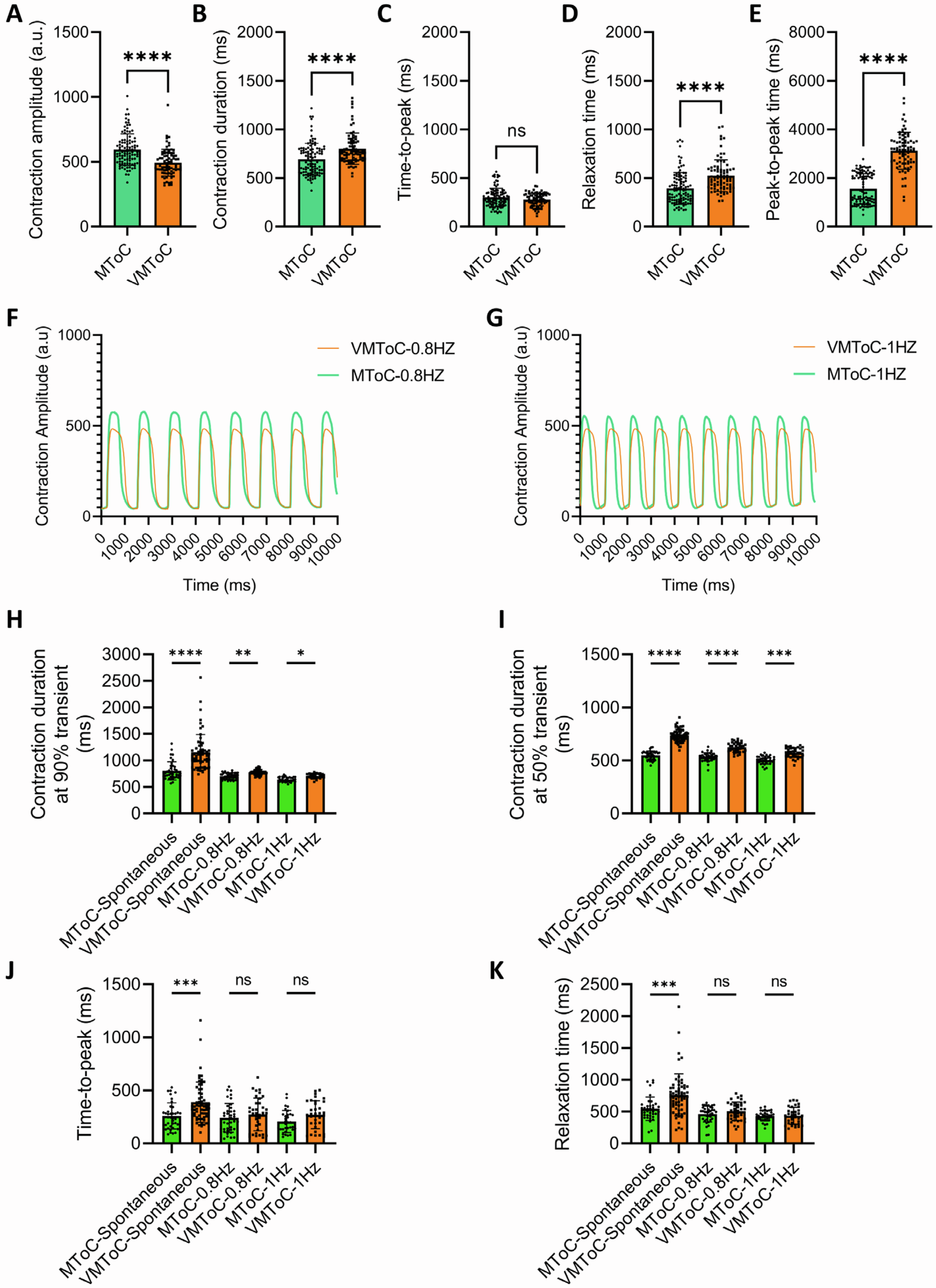

A
B
C
D
E
Contraction amplitude (a.u.)
Contraction duration (ms)
Time-to-peak (ms)
Relaxation time (ms)
Peak-to-peak time (ms)
****
ns
MToC
VMToC
F
G
Contraction Amplitude (a.u)
VMToC-0.8HZ
MToC-0.8HZ
VMToC-1HZ
MToC-1HZ
Time (ms)
H
I
Contraction duration at 90% transient (ms)
Contraction duration at 50% transient (ms)
J
K
MToC-Spontaneous
VMToC-Spontaneous
MToC-0.8Hz
VMToC-0.8Hz
MToC-1Hz
VMToC-1Hz

Figure S3. Characterization of contraction parameters upon pacing. Related to Figure 3. (A-E) Quantification of the contraction parameters of MToC and VMToC: contraction amplitude (A); contraction duration (B); time-to-peak (C); relaxation time (D); peak-to-peak time (E) in MTs with LUMC0059iCTRL03 hiPSC-CMs. Error bars are shown as ±SD from MToC N = 3, n>30; VMToC N =3, n>26; three independent experiments with 26 MTs from at least six different microfluidic channels in each experiment. (F-G) Representative beating traces at 0.8 Hz (A) and 1 Hz (B). (H-K) Quantification of the contraction parameters during spontaneous beating, at 0.8 Hz and 1 Hz paced MTs: contraction duration at 90% transient (H); contraction duration at 50% transient (I); time-to-peak (J); relaxation time (K) in MTs with AICS-0075 hiPSC-CMs. Error bars are shown as ±SD from MToC N = 3, n >10; VMToC N =3, n>10; three independent experiments with 10 MTs from at least three different microfluidic channels were carried out for each experiment.

Wilcoxon-Mann-Whitney test (A-E), Kruskal-Wallis test with Dunn's multiple comparisons test (H-K); $*p < 0.05$, $**p < 0.01$; $***p < 0.001$, $****p < 0.0001$; ns, not significant.

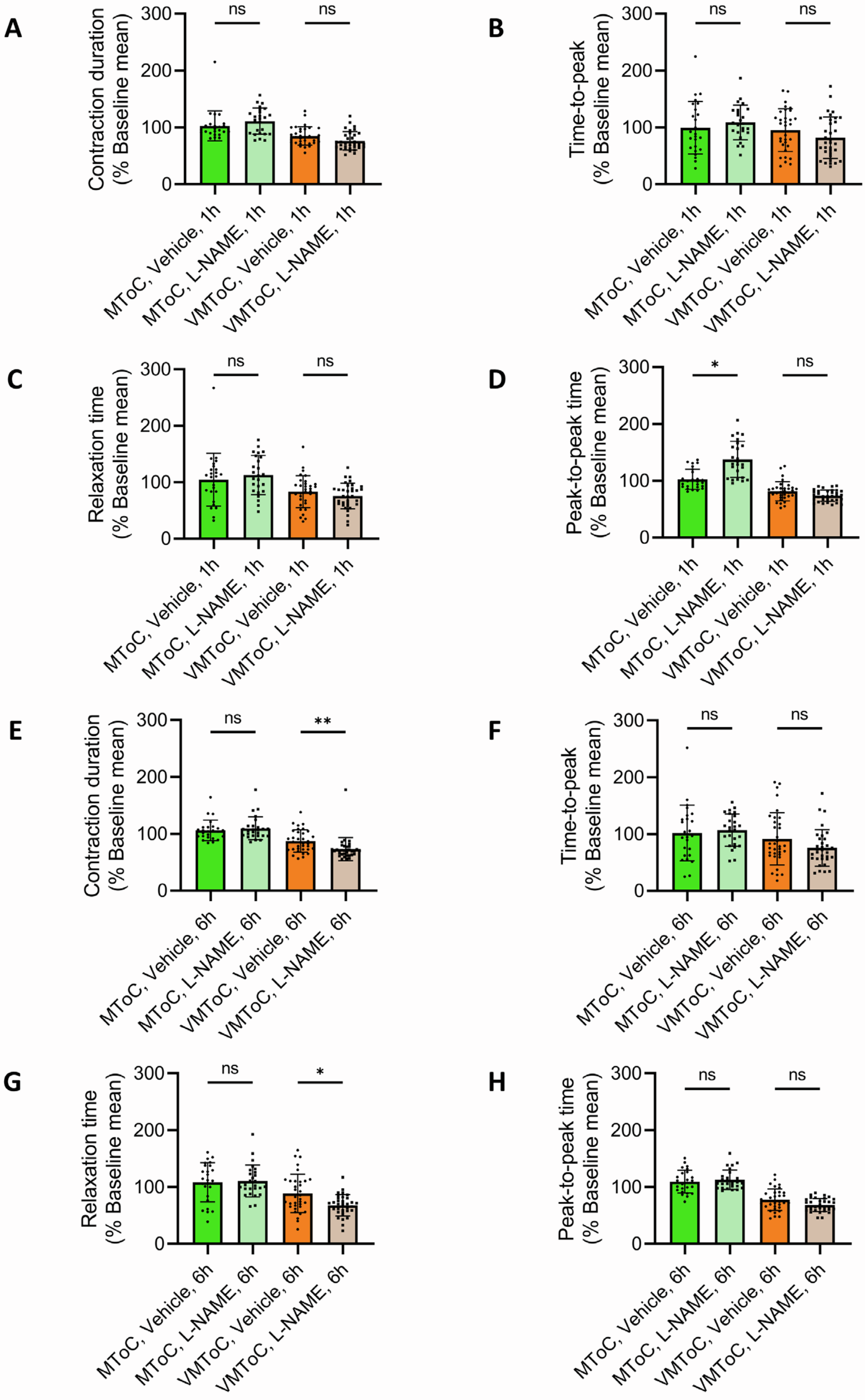
A
B
C
D
E
F
G
H
Contraction duration (% Baseline mean)
Time-to-peak (% Baseline mean)
Relaxation time (% Baseline mean)
Peak-to-peak time (% Baseline mean)
0
100
200
300
ns
*
**
MToC, Vehicle, 1h
MToC, L-NAME, 1h
VMToC, Vehicle, 1h
VMToC, L-NAME, 1h
MToC, Vehicle, 6h
MToC, L-NAME, 6h
VMToC, Vehicle, 6h
VMToC, L-NAME, 6h

Figure S4: Altered contractile dynamics of cardiac MTs upon inhibition of EC-CM communication in VMToC but not in MToC. Related to Figure 4. (A-H) Quantification of the contraction parameters presented as percentage change from the baseline mean of spontaneous beating condition, after 1 h (A-D) and 6 h (E-H) incubation of L-NAME (1 mM): contraction duration (A, E); time-to-peak (B, F); relaxation time (C, G); peak-to-peak time (D, H) in MTs with AICS-0075 hiPSC-CMs. Error bars are shown as ±SD from MToC N = 3, n >6; VMToC N =3, n > 7; three independent experiments with at least 6 MTs from at least four different microfluidic channels in each experiment. Data is normalized to baseline mean of each set for vehicle and L-NAME group of MTs.
Kruskal-Wallis test with Dunn’s multiple comparisons test (A-H). $*p < 0.05$, $**p < 0.01$; ns, not significant.

Supplemental Tables:

Table S1. List of hiPSC lines and cell batches used in this study.

**Figures**	**hiPSC-CM**		**hiPCS-EC (internal vascular network)**		**hiPSC-EC (external vascular network)**		**hiPSC-CF**	
	**Cell Line**	**Number of batches**	**Cell Line**	**Number of batches**	**Cell Line**	**Number of batches**	**Cell Line**	**Number of batches**
Figure 1, 3, S1, S3F-K, S4, Video S1, S2, S3	AICS-0075	3	NCRM-1 (mCherry)	3	NCRM-1 (mCherry)	4	LUMC0020 iCTRL06	3
Figure 2B-E, 2G, 2F (Line 2), S2	LUMC0020 iCTRL06	3	NCRM-1 (GFP)	1	NCRM-1 (mCherry)	4	LUMC0020 iCTRL06	3
Figure 2F	AICS-0075 (Line 1); LUMC0020 iCTRL06 (Line 2); LUMC0059 iCTRL03 (Line 3)	1<br>1<br>1	NCRM-1 (mCherry)	1	NCRM-1 (mCherry)	1	LUMC0020 iCTRL06	1
Figure 2F (line 3), 4, S3A-E	LUMC0059 iCTRL03	1	NCRM-1 (mCherry)	1	NCRM-1 (mCherry)	1	LUMC0020 iCTRL06	1

Supplemental Experimental Setup

hiPSC lines and maintenance

hiPSCs were maintained on recombinant vitronectin-coated plates in TeSR-E8, all from STEMCELL Technologies, according to the manufacturer's instructions. The following hiPSC lines were used in this study: LUMC0059iCTRL03 (generated from skin fibroblasts, https://hpscreg.eu/cell-line/LUMCi026-A), LUMC0020iCTRL06 (generated from skin fibroblasts, https://hpscreg.eu/cell-line/LUMCi028-A) (Zhang et al., 2014); NIH Center for Regenerative Medicine: NCRM-1 (generated from CD34+ cord blood cells, https://hpscreg.eu/cell-line/CRMi003- A), obtained from RUDCR Infinite Biologics at Rutgers University, was modified in-house with a mCherry or GFP expression cassette under the human cytomegalovirus (hCMV) early enhancer/chicken β actin (CAG) promoter using a previously established protocol (Rostovskaya et al., 2012); The Allen Cell Collection: AICS-0075 (generated from skin fibroblasts, https://hpscreg.eu/cell-line/UCSFi001-A-4) with mEGFP insertion site at ACTN2 was obtained from Coriell Institute for Medical Research. hiPSC lines and cell batches of hiPSC-CMs, hiPSC-ECs and hiPSC-CFs are listed in Table 1.

Differentiation of cardiomyocytes

CM differentiation of hiPSC was induced in monolayer as described previously (Berg et al., 2016; Giacomelli et al., 2017). Briefly, 25 × 10^3 cells per cm2 were seeded on plates coated with 75 μg/ml growth factor-reduced Matrigel (Corning) the day before the start of differentiation (day -1). On day 0, cardiac mesoderm was induced by changing E8 to B(P)EL medium (Bovine Serum Albumin [BSA] and Essential Lipids), supplemented with a cytokine mixture (20 ng/ml BMP4, R&D Systems; 20 ng/ml ACTIVIN A, Miltenyi Biotec; 1.5 μM GSK3 inhibitor CHIR99021, Axon Medchem). After 3 days, cytokines were removed and XAV939 which is WNT inhibitor (5 μM, Tocris) was added for 3 days. After this, B(P)EL medium was refreshed every 3 days.

Differentiation of cardiac fibroblasts

CFs were differentiated from hiPSC using an epicardial (EPI) monolayer differentiation protocol (Guadix et al., 2017) as a starting point (Giacomelli et al., 2020). Briefly, 25 × 10^3 cells per cm2 were seeded on Matrigel at day -1. On day 0, cardiac mesoderm was induced as described above. After 3 days, cytokines were removed and XAV939 (5 μM) was added for 3 days with BMP4 (30 ng/ml) and Retinoic Acid (RA; 1 μM; Sigma Aldrich). On day 6, B(P)EL medium supplemented with BMP4 (30 ng/ml) and RA (1 μM) was refreshed. On day 9, 15 × 10^3 per cm2 were seeded on plates coated with 5 μg/ml of fibronectin from bovine plasma (fibronectin; Sigma Aldrich) in B(P)EL medium supplemented with the TGFβ inhibitor SB431542 (10 μM; Tocris). By day 12, EPIs were confluent and ready for passaging or analysis. EPI cells (30 cm2 per vial) were cryopreserved in CryoStor CS10 medium (0.5 ml/vial; Stem Cell Technologies).

CF differentiation was induced in monolayer. Briefly, 25 × 10^3 EPIs were seeded per cm2 on tissue culture plates coated with vitronectin in B(P)EL medium supplemented with FGF2 (10 ng/ml; R&D Systems) on day 12. On day 13 and every 2 days thereafter, medium was refreshed with B(P)EL supplemented with FGF2 (10 ng/ml). After 6 days (on day 19), CFs were expanded by changing B(P)EL to Fibroblast Growth Medium 3 (FGM3; PromoCell). FGM3 was refreshed every 2 days for approximately 10 days in total. After 10 days (on day 29), CFs were confluent and ready to be passaged at 1:2 ratio. FGM3 was refreshed the day after passaging and every 2 days thereafter. CFs (10cm2 per vial) were cryopreserved in CryoStor CS10 medium (0.5 ml/vial; Stem Cell Technologies).

Differentiation of endothelial cells

hiPSC differentiation to ECs was performed as described previously (Orlova et al., 2014). Briefly, hiPSCs were maintained in mTeSR-E8 medium. For mesoderm induction (day 0-3), mTeSR-E8 medium was replaced with B(P)EL medium supplemented with 8 μM CHIR99021 (Tocris Bioscience, 4423). Cells were refreshed with vascular specification medium comprised of VEGF (50 ng/ml) and 10 μM SB431542 (Tocris Bioscience, 1614) in B(P)EL at day 3, day 6, and day 9. hiPSC-ECs were isolated on day 10 using CD31-Dynabeads™ (Thermo Fisher Scientific), as previously described (Orlova et al., 2014). hiPSC-ECs were expanded in complete EC growth medium (EC-CGM) comprised of Human Endothelial-serum free medium (EC-SFM) with 1% Human platelet poor serum (P2918, Sigma), VEGF (30 ng/ml) and bFGF (20 ng/ml), as described previously with minor modifications (Orlova et al., 2014). hiPSC-ECs were expanded for additional 3-4 days post-isolation and cryopreserved using cryopreservation medium consists of 50% fetal bovine serum,40% EGM2 and 10% dimethyl sulfoxide at passage number 1 (P1) (StemCell Technologies, 07930).

3D cardiac microtissue formation

MTs were formed as described previously (Giacomelli et al., 2020). Prior to MT formation, hiPSC-ECs and hiPSC-CFs were prepared as follows: 3-4 days before MT formation, a vial of cryopreserved hiPSC-ECs and a vial of cryopreserved hiPSC-CFs were thawed and cultured either in EC-CGM on plates coated with gelatin (hiPSC-ECs), or in FGM3 on uncoated plates (hiPSC-CFs). On the day of MT formation (day 0), hiPSC-ECs and hiPSC-CFs were detached using TrypLE 1X for 2-3 mins at RT (EC) and 5 mins at 37 °C, 5 % CO2 (CF), centrifuged for 3 min at 1100 rpm, resuspended in B(P)EL medium and counted. hiPSC-CMs at day 14-21 that showed > 80 % purity, measured as the percentage of troponin positive cells by FACS, were dissociated using the TryplE 5X for 10 mins at 37 °C, 5 % CO2, resuspended in B(P)EL medium and counted. Cell suspensions were combined to a total of 5000 cells (70 % CM, 15 % EC and 15 % CF) per 50 µl B(P)EL medium supplemented with VEGF (50 ng/ml) and FGF2 (5 ng/ml). For all MTs, cell suspensions were seeded on V-bottomed 96-well microplates (Greiner bio-one) and centrifuged for 10 min at 1100 rpm. MTs were incubated at 37 °C, 5 % CO2 for 12 days with media refreshed every 3-4 days.

Cell Preparation before on chip culture

Human brain vascular pericytes (HBVPs) were purchased from ScienceCell. HBVPs were cultured in Pericyte Medium (ScienceCell, 1201) supplemented with Pericyte Growth Supplement (ScienceCell, 1252) and 2 % FBS. Cells were cryopreserved at passage number 3 (P3) using serum-free cryopreservation medium (CryoStor™CS10) (StemCell Technologies, 07930). hiPSC-ECs (P1) were thawed and cultured on gelatin-coated plates in EC-CGM, 4 days prior to VoC seeding. HBVPs (P3) were thawed and cultured in Pericyte Medium (ScienceCell, 1201) supplemented with Pericyte Growth Supplement (ScienceCell, 1252) and 2 % FBS.

L-NAME and IL-1β stimulation

On the day of L-NAME stimulation chips were refreshed with the medium supplemented with either VEGF (50 ng/mL) and distilled water (Vehicle) or VEGF (50 ng/mL) and L-NAME (1 mM, N5751, Sigma) for one or six hours. Chips were incubated at 37 °C, 5% CO2 and contraction analyses were performed after one hour or six hours.

On the day of IL-1β stimulation chips were refreshed with the medium supplemented with either VEGF (50 ng/mL) and PBS (Vehicle) or VEGF (50 ng/mL) and IL-1β (10 ng/ml, N5751, Sigma) for twelve hours. Chips were incubated at 37 °C, 5% CO2 and contraction analyses were performed after twelve hours.

Multiplex Cytokine Analysis

The medium from multiple chips were collected after twelve hours stimulation with IL-1β (10 ng/ml) or vehicle, and kept at -80 ºC. On the day of the multiplex cytokine bead assay, collected medium was thawed two hours prior to the experiment. Concentration of cytokines (CXCL10, IL-1β, TNFα, CCL2, IL-6, IL-10, IFNϒ, TGFβ1 free active and IL-8) was measured using a LEGENDplex Human Essential Immune Response Panel kit (13-plex) (BioLegend, cat no: 740930) according to the manufacturer's instructions. Undiluted or eight times diluted samples were run on Cytek 3-Laser Aurora spectral flow cytometer (Cytek Biosciences, USA).

Imaging of vascularized 3D cardiac microtissues on chip

Whole channel images were captured daily with EVOSM7000 using 10x objectives. For automated imaging of whole channels and stitching, a customized plate layout was used. VMToCs and MToCs were fixed for 30 mins at 4 °C with 4% paraformaldehyde, washed 3 times in PBS (Calcium, Magnesium) and stored at 4 °C until processing. Confocal images were captured to create 3D stack using Andor Dragonfly spinning disk confocal microscope using a 20x or 40x objective. Image processing was done using Imaris 9.5 software (Bitplane, Oxford Instruments).

Characterization of vascular networks

Fields of views (FOV) from the full channels (acquired using EVOS) were arranged in size of 480X1200 pixels around the MTs. For VoC 75 FOVs and for VMToC 87 FOVs were captured from three independent experiments with at least six microfluidic channels each. These FOVs (Figure 1E-H), whole microfluidic channels (Figure S2D), and 10x images of MTs from channels (Figure S2E) were then quantified using pipelines developed on the free open source CellProfiler software (https://cellprofiler.org/) (Carpenter et al., 2006; Vila Cuenca et al., 2021). Briefly, for vessel density, pre-processing steps were applied to all images to enhance image features and a gaussian filter to reduce unspecific object identification. Two filter steps were applied to images of vascular network to

reduce non-specific segmentation from cell junctions and a minimum cross-entropy thresholding method was used to produce a binarized image. The binarized images from the CellProfiler output were then analyzed using the freely available ImageJ software with a plugin (https://imagej.nih.gov/ij/ ,https://imagej.net/DiameterJ) (Hotaling et al., 2015).

Contraction analysis

For pacing experiments, electrodes were made in-house to fit to the gel channels of chips and used to pace MTs. MTs were stimulated at 0.8 and 1 Hz, with 15V/cm strength and 3 ms long stimulation pulse. Movies of spontaneous beating or paced MTs from VMToC and MToC conditions were acquired for at least 10 s at 37 °C either with a ThorLabs DCC3240M camera at 100 frames/s and a 10x objective phase contrast objective (Leica Inverted microscope IBDE), using the ThorLabs uc480 software (v 4.20), or with a Leica Microsystems LAS AF6000 microscope at 37 °C and 5 % CO2. On the day of L-NAME or IL-1β stimulation, VMToC and MToC conditions were recorded for their baseline spontaneous beating prior to drug administration. Chips were recorded after both one hour incubation and six hours incubation period with vehicle and L-NAME or twelve hours incubation period with IL-1β. Contraction data were obtained by analyzing movies with the MUSCLEMOTION ImageJ macro (ImageJ v. 2.0.0-rc-49) as described previously (Sala et al., 2018).

Sarcomere analysis

Confocal images of VMToC and MToC conditions were acquired using a DragonFly spinning disk (Andor) microscope with 40x objective. In each MT, one plane from each FOVs were chosen to quantify and converted to 8-bit images. For MToC, 88 FOVs were selected in 11 MTs and for VMToC 52 FOVs were selected in 8 MTs. Sarcomere quantification data was obtained by analyzing these 8-bit images with SotaTool, as described previously (Stein et al., 2022).

Fluorescent beads perfusion assay

Perfusion assessment of VMToC was done as described previously (Vila Cuenca et al., 2021). In brief, 405-beads (1 drop in 10 or 20 ml EGM-2, Fluoro-Max Dyed Blue Aqueous Fluorescent Particles, B0200, ThermoFisher Scientific) were added to the right medium port (100 μl) and to all other media ports (50 μl). Then, confocal image stacks and videos of the spontaneous move of the beads following the endothelial networks were acquired using DragonFly spinning disk (Andor) microscope with 20x and 40x magnification objective. Tracking of the beads was done using 3D rendering and cell tracking function in Imaris 9.5 software (Bitplane, Oxford Instruments).

Supplemental References

Berg, C.W. van den, Elliott, D.A., Braam, S.R., Mummery, C.L., and Davis, R.P. (2016). Differentiation of Human Pluripotent Stem Cells to Cardiomyocytes Under Defined Conditions. Methods Mol. Biol. *1353*, 163–180.

Carpenter, A.E., Jones, T.R., Lamprecht, M.R., Clarke, C., Kang, I.H., Friman, O., Guertin, D.A., Chang, J.H., Lindquist, R.A., Moffat, J., et al. (2006). CellProfiler: image analysis software for identifying and quantifying cell phenotypes. Genome Biol. *7*, R100.

Giacomelli, E., Bellin, M., Orlova, V. V, and Mummery, C.L. (2017). Co-Differentiation of Human Pluripotent Stem Cells-Derived Cardiomyocytes and Endothelial Cells from Cardiac Mesoderm Provides a Three-Dimensional Model of Cardiac Microtissue. Curr. Protoc. Hum. Genet. *95*, 21.9.1-21.9.22.

Giacomelli, E., Meraviglia, V., Campostrini, G., Cochrane, A., Cao, X., van Helden, R.W.J., Krotenberg Garcia, A., Mircea, M., Kostidis, S., Davis, R.P., et al. (2020). Human-iPSC-Derived Cardiac Stromal Cells Enhance Maturation in 3D Cardiac Microtissues and Reveal Non-cardiomyocyte Contributions to Heart Disease. Cell Stem Cell *26*, 862-879.e11.

Guadix, J.A., Orlova, V. V, Giacomelli, E., Bellin, M., Ribeiro, M.C., Mummery, C.L., Pérez-Pomares, J.M., and Passier, R. (2017). Human Pluripotent Stem Cell Differentiation into Functional Epicardial Progenitor Cells. STEMCR *9*, 1754–1764.

Hotaling, N.A., Bharti, K., Kriel, H., and Simon, C.G. (2015). DiameterJ: A validated open source nanofiber diameter measurement tool. Biomaterials *61,* 327-338.

Orlova, V. V., Van Den Hil, F.E., Petrus-Reurer, S., Drabsch, Y., Ten Dijke, P., and Mummery, C.L. (2014). Generation, expansion and functional analysis of endothelial cells and pericytes derived from human pluripotent stem cells. Nat. Protoc. *9,* 1514-1531

Rostovskaya, M., Fu, J., Obst, M., Baer, I., Weidlich, S., Wang, H., Smith, A.J.H., Anastassiadis, K., and Francis Stewart, A. (2012). Transposon-mediated BAC transgenesis in human ES cells. Nucleic Acids Res. *40,* e150.

Sala, L., van Meer, B.J., Tertoolen, L.G.J., Bakkers, J., Bellin, M., Davis, R.P., Denning, C., Dieben, M.A.E., Eschenhagen, T., Giacomelli, E., et al. (2018). Musclemotion: A versatile open software tool to quantify cardiomyocyte and cardiac muscle contraction in vitro and in vivo. Circ. Res. *122,* e5-e16.

Stein, J.M., Arslan, U., Franken, M., De Greef, J.C., Harding, S.E., Mohammadi, N., Orlova, V. V, Bellin, M., Mummery, C.L., and Van Meer, B.J. (2022). Software Tool for Automatic Quantification of Sarcomere Length and Organization in Fixed and Live 2D and 3D Muscle Cell Cultures In Vitro. Curr. Protoc. Hum. Genet. *2,* e462.

Vila Cuenca, M., Cochrane, A., van den Hil, F.E., de Vries, A.A.F., Lesnik Oberstein, S.A.J., Mummery, C.L., and Orlova, V. V. (2021). Engineered 3D vessel-on-chip using hiPSC-derived endothelial- and vascular smooth muscle cells. Stem Cell Reports *16,* 2159-2168.

Zhang, M., D'Aniello, C., Verkerk, A.O., Wrobel, E., Frank, S., Ward-Van Oostwaard, D., Piccini, I., Freund, C., Rao, J., Seebohm, G., et al. (2014). Recessive cardiac phenotypes in induced pluripotent stem cell models of Jervell and Lange-Nielsen syndrome: Disease mechanisms and pharmacological rescue. Proc. Natl. Acad. Sci. U. S. A. *111,* e5383-e5392.
