# Supplementary material for: Vascularized hiPSC-derived 3D cardiac microtissue on chip
Source: Stem Cell Reports. 2023 Jun 29;18(7):1394–404. doi: 10.1016/j.stemcr.2023.06.001 (PMC10362508; doi:10.1016/j.stemcr.2023.06.001)
Supplement: Document S2. Article plus supplemental information [file mmc5.pdf]

Stem Cell Reports
Report
OPEN ACCESS

Vascularized hiPSC-derived 3D cardiac microtissue on chip

Ulgu Arslan,[1] Marcella Brescia,[1] Viviana Meraviglia,[1] Dennis M. Nahon,[1] Ruben W.J. van Helden,[1] Jeroen M. Stein,[1] Francijna E. van den Hil,[1] Berend J. van Meer,[1] Marc Vila Cuenca,[1,2] Christine L. Mummery,[1] and Valeria V. Orlova[1,*]
[1]Department of Anatomy and Embryology, Leiden University Medical Centre, 2333ZC Leiden, the Netherlands
[2]Department of Clinical Genetics, Leiden University Medical Center, 2333ZA Leiden, the Netherlands
*Correspondence: v.orlova@lumc.nl
https://doi.org/10.1016/j.stemcr.2023.06.001

SUMMARY

Functional vasculature is essential for delivering nutrients, oxygen, and cells to the heart and removing waste products. Here, we developed an *in vitro* vascularized human cardiac microtissue (MT) model based on human induced pluripotent stem cells (hiPSCs) in a microfluidic organ-on-chip by coculturing hiPSC-derived, pre-vascularized, cardiac MTs with vascular cells within a fibrin hydrogel. We showed that vascular networks spontaneously formed in and around these MTs and were lumenized and interconnected through anastomosis. Anastomosis was fluid flow dependent: continuous perfusion increased vessel density and thus enhanced the formation of the hybrid vessels. Vascularization further improved endothelial cell (EC)-cardiomyocyte communication via EC-derived paracrine factors, such as nitric oxide, and resulted in an enhanced inflammatory response. The platform sets the stage for studies on how organ-specific EC barriers respond to drugs or inflammatory stimuli.

INTRODUCTION

Cardiovascular disorders are a major cause of death around the world. The heart is a highly metabolic organ and has high energy demands for proper function. Endothelium is crucial in this process as it forms a semi-permeable barrier between cardiomyocytes (CMs) and the blood, providing selective nutrient, oxygen, and drug delivery to heart cells. It also mediates immune cell trafficking, for example, in the case of inflammation (Amersfoort et al., 2022). Interruption of the blood supply can lead to CM death, as in myocardial infarction (Berry and Duncker, 2020), and successful heart transplantation requires rapid restoration of blood supply to the donor heart.

Several 3D microphysiological models of the human heart have been described in which cell-cell crosstalk improved human pluripotent stem cell derived CM (hPSC-CM) maturation. These systems have proven useful for modeling some types of cardiac disease and for drug screening (reviewed in Arslan et al., 2022). Some of these models incorporated vascular networks inside engineered tissues, but in general, they were not perfusable by fluid or blood (equivalents) (Zhang et al., 2021). This has in part been addressed by either integrating organoids into a microfluidic chip or implanting them into living animals, as perfusion is also necessary for the stability and integrity of the microvascular networks (Mansour et al., 2018; Ryan et al., 2021; Takebe et al., 2013).

In the study here, we developed a fully vascularized and perfusable cardiac microtissue (MT) on-chip by integrating pre-vascularized cardiac MTs with an external vascular network formed by self-organization of human induced pluripotent stem cell-derived endothelial cells (hiPSC-ECs) and human brain vascular pericytes (HBVPs) in fibrin hydrogel in a microfluidic organ-on-chip. The integration of MTs in the chips did not adversely affect formation of the external, self-organized vascular network, and these networks developed robustly inside and around the MTs. Using this model, we demonstrated that cardiac MTs can be efficiently vascularized within days, and the lumenized vascular networks in and around the MTs can be perfused. This may ultimately allow selective nutrient or drug delivery to the cells via the endothelial barrier inside the MTs, mimicking heart tissue *in vivo* even more closely. Notably, we demonstrated that EC-CM crosstalk can be modeled in vascularized cardiac MT on chip (VMToC) cultures. By using either L-N-nitro arginine methyl ester (L-NAME), an inhibitor of nitric oxide synthetases (NOSs), or pro-inflammatory stimulus, such as interleukin-1β (IL-1β), we revealed relevant paracrine signals playing roles in the regulation of cardiac function in MTs.

RESULTS

Characterization of the vascular network in the presence of cardiac MTs

The VMToC model was generated by combining pre-vascularized cardiac MTs (Giacomelli et al., 2020; Campostrini et al., 2021) with hiPSC-ECs and HBVPs in a fibrin hydrogel. This MT/cell/hydrogel mix was then embedded in the gel channel of commercially available AIM Biotech 3D cell culture chips (Figure 1A). To investigate the effect of cardiac MTs on vascular network formation, we compared the VMToC model with a standard vessel-on-chip (VoC) that included hiPSC-EC and HBVP cocultures but without cardiac MTs (Figure 1B). Vascular organization in VoCs and

 © 2023 The Author(s).
This is an open access article under the CC BY license (http://creativecommons.org/licenses/by/4.0/).

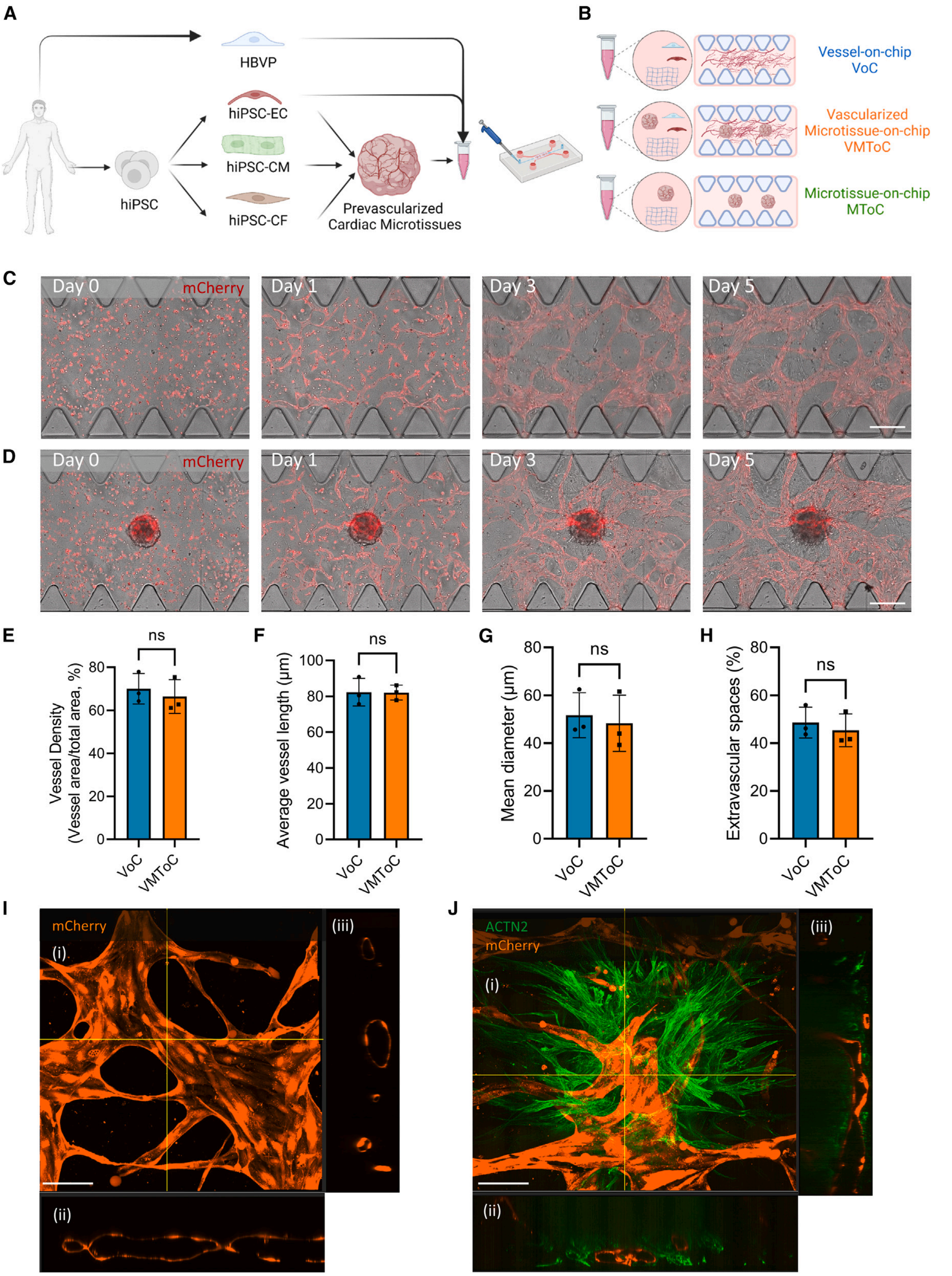

(legend on next page)

VMToCs was already evident on day 1 (Figures 1C and 1D). On day 3, a complex vascular network had formed in the chips, and these networks were stabilized by day 5. Quantification of the vascular network parameters such as vessel density (Figure 1E), average vessel length (Figure 1F), mean vessel diameter (Figure 1G), and extravascular space (Figure 1H) showed no significant differences between VoC and VMToC conditions. Vascular networks in VoC (Figure 1I) and the microvasculature in MTs in VMToC (Figure 1J, Video S1) were both lumenized. To investigate whether the lumenized vasculature in cardiac MTs was actually perfusable, fluorescent beads (2 μm) were introduced into the medium in one of the media inlets of the chips. Beads entered the vascular networks from the intersection of medium-gel channel and moved spontaneously to the microvasculature in MTs (Figure S1A). Beads stayed within the microvasculature contours in the MTs indicating the interconnected vessels in the MTs were indeed perfusable (Figures S1B–S1E, Videos S2 and S3).

Mechanism of the intra-microvascular network formation

In order to investigate the mechanism of the intra-microvascular network formation, we used hiPSC-ECs derived from two different fluorescent reporter lines (expressing mCherry or GFP). GFP-expressing hiPSC-ECs were used to prevascularize the cardiac MTs, and these MTs were cocultured with mCherry-expressing hiPSC-ECs and HBVPs in the chips (Figure 2A). Internal microvasculature in the MTs and external vascular network around MTs underwent anastomosis as early as day 2. Anastomosis was bidirectional in the system: (1) outside-in anastomosis where the external network invaded the MTs and anastomosed with the internal microvasculature in the MTs (Figures 2B and 2D) and (2) inside-out anastomosis where part of the internal microvascular network migrated outside the MT and anastomosed with the external vascular network (Figures 2C and 2E). As a result, hybrid vessels composed of hiPSC-ECs partly from MTs and partly from external vascular networks were formed. Confocal images showed that these hybrid vessels were lumenized (Figures 2D and 2E). The number of vascularized tissues was comparable in all independent experiments (Figure 2F), with higher numbers of MTs showing evidence of outside-in anastomosis than those showing inside-out anastomosis (Figure 2G).

Since fluid flow is known to promote EC migration, proliferation, and survival (Abe et al., 2019; Galie et al., 2014; Zhang et al., 2022), we tested whether we could enhance the vascularization of cardiac MTs by introducing perfusion using a rocker (Figure S2A). Both intermittent and continuous perfusion resulted in anastomosis between the internal microvasculature and external vascular networks (Figures S2B and S2C, respectively). However, continuous perfusion resulted in higher mCherry+ and GFP+ vessel density (Figures S2D and S2E) in the chips. Intermittent perfusion resulted in the formation of hybrid vessels where only a minority of cells originated in the cardiac MTs (Figure S2F). This improved substantially under continuous perfusion (Figure S2G).

Characterization of cardiac MTs in the absence or presence of external vascular network

We next examined cardiac MTs in the chips for any changes in their sarcomere organization and contractile properties. MTs were compared in the absence (MToC, Video S1, Figures 1B and 3A) or presence (VMToC, Figure 3B) of an external vascular network. Sarcomere morphologies appeared similar in the MToC (Figure 3C) and VMToC (Figure 3D), which was confirmed by similar sarcomere lengths (Figure 3E) and alignment indices (Figure 3F) assessed using the SOTATool (Stein et al., 2022). This indicated that there was no additional effect on sarcomere organization in the presence of external vascular network.

Contractility of cardiac MTs was analyzed using the video-based software tool MUSCLEMOTION (Sala et al., 2018). Representative beating traces showed that MTs under both conditions maintained their contractility in

Figure 1. Characterization of the vascular network in the presence of cardiac MTs

(A and B) Schematic overview of the vascularized cardiac MTs experimental setup (A) and conditions used in the study (B). hiPSC-ECs and HBVPs were cocultured to form VoC; hiPSC-MTs were cocultured with hiPSC-ECs and HBVPs to form VMToC; hiPSC-MTs were integrated into chips without any additional vascular cells to form MToC.

(C and D) Representative images from chips on days 0, 1, 3, and 5 after seeding in the chips, which shows the development of external vascular networks in VoC (C) and VMToC (D). Images showing bright field and hiPSC-EC (red, mCherry) (10×). Scale bars, 300 μm.

(E–H) Quantification of vessel density (%) (E), average vessel length (μm) (F), mean diameter (μm) (G), extravascular spaces (%) (H). Error bars are shown as mean ± SD from N = 3; three independent experiments with at least six microfluidic channels per experiment. Student's t test; ns, not significant.

(I and J) Representative confocal images of microvascular network in VoC (I) and VMToC (J) showing hiPSC-ECs (orange, mCherry) and hiPSC-CMs (green, ACTN2). Images displaying maximum projection in xyz (i), xy (ii), and yz cross-sectional perspectives (iii) (40×). Scale bar, 100 μm.

See also Figure S1 and Videos S1, S2, and S3.

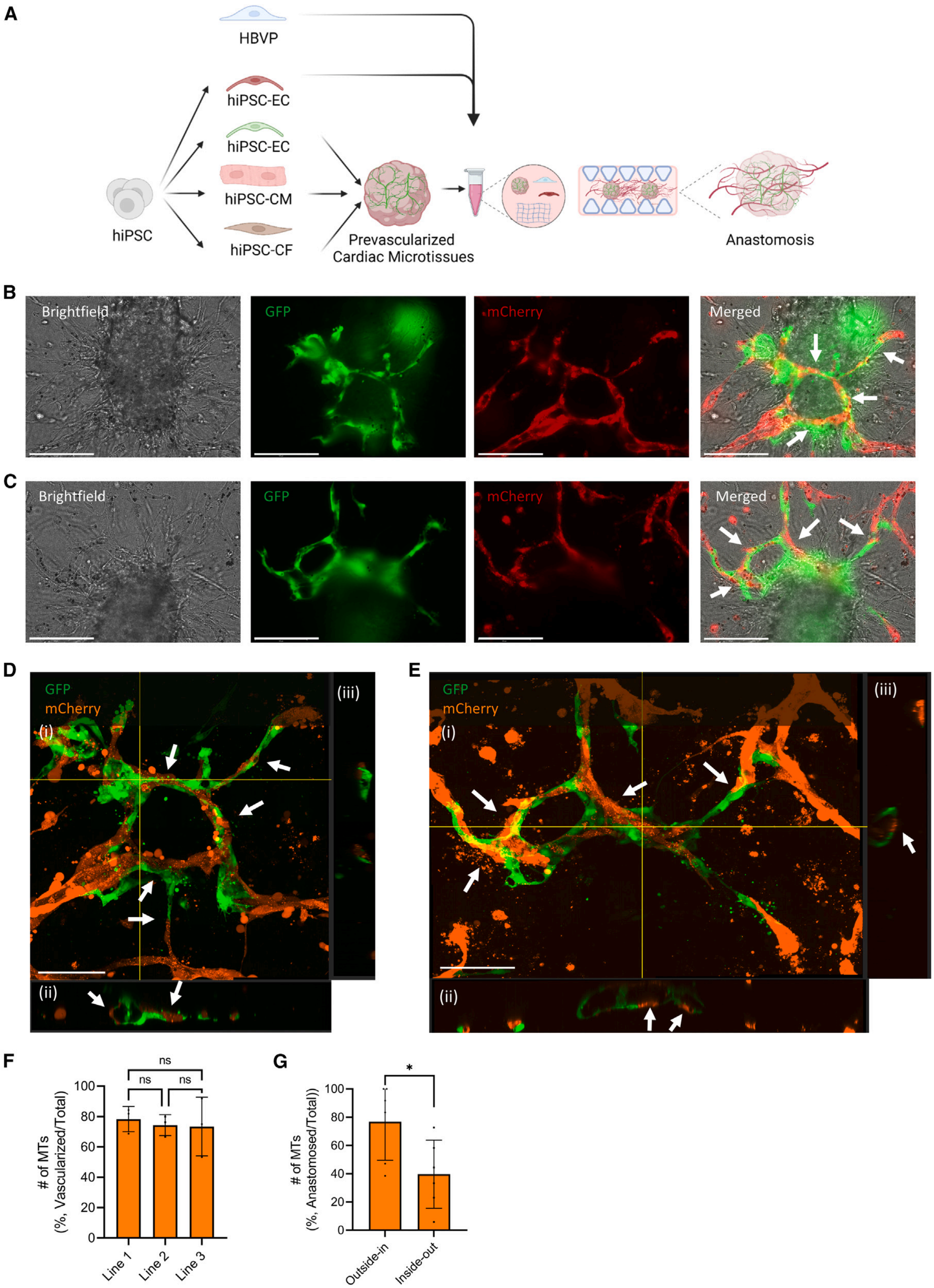

(legend on next page)

hydrogel (Figure 3G). However, contraction amplitude (Figure 3H) was significantly lower in VMToC, and contraction duration (Figure 3I), relaxation time (Figure 3K) and peak-to-peak time (Figure 3L) were significantly longer in VMToC. Time-to-peak (Figure 3J) was similar in both conditions. The changes in these parameters were consistent in VMToC and MToC that were generated using a second, independent hiPSC line for CMs (Figures S3A–S3E). In order to test whether the increase in contraction duration and relaxation time in the VMToC is not rate dependent, we performed electrical pacing in the chips using custom-made electrodes that fit the gel channel inlet and outlet. When these MTs were paced at 0.8 Hz and 1 Hz (Figures S3F and S3G), contraction duration at 90% and 50% transient was significantly higher in VMToC compared with MToC, consistent with the results under conditions of spontaneous beating (Figures S3H and S3I). We further investigated if the duration differences could be explained by time-to-peak or relaxation time changes. However, these parameters were highly variable and were not significantly different between VMToC and MToC even when paced (Figures S3J and S3K).

Modeling EC-CM crosstalk in cardiac MTs

To explore whether VMToC has added value over MToC in studying inflammation, we investigated whether EC-CM crosstalk can be modulated by either (1) L-NAME, a nonselective inhibitor of NOS, or (2) a pro-inflammatory stimulus, such as IL-1β. MToC and VMToC were incubated with L-NAME or vehicle for 1 h (Figures 4A–4D and S4A–S4D) or 6 h (Figures 4E–4H and S4E–S4H). L-NAME had no effect on the contraction time parameters: duration, time to peak, and relaxation time in MToC after 1- or 6-h incubation compared with the vehicle alone. By contrast, 6-h L-NAME exposure of VMToC cultures decreased contraction duration (Figures 4E and S4E) and relaxation time (Figures 4G and S4G) and had no effect on the time to peak (Figures 4F and S4F). 1 h of L-NAME incubation was more variable between the lines tested and decreased contraction duration and relaxation time in one but not the other control line (Figures 4A, 4C, S4A, and S4C). Peak-to-peak times appeared to decrease in one of the control lines tested after 1 and 6 h but not the other (Figures 4D, 4H, S4D, and S4H).

Finally, MToC and VMToC were stimulated for 12 h with IL-1β (10 ng/mL) to investigate the effect of pro-inflammatory cytokines. Pro-inflammatory cytokine release and contractile parameters were assessed. Stimulation with IL-1β resulted in significant upregulation of IL-6 (Figure 4I), IL-8 (Figure 4J), and cytokine chemoattractant protein 1 (MCP-1/CCL2, Figure 4K) in VMToC but not in MToC. In addition, IL-1β stimulation decreased contraction duration, relaxation time, and peak-to-peak time in VMToC but had no effect on MToC (Figures 4L–4O).

DISCUSSION

In the present study, we established vascularized and perfusable cardiac MT on a VMToC with vascular cells self-organized in fibrin hydrogel. The external vascular cells reproducibly formed a continuous vascular network in and around the MTs, and this was not affected by contraction of the MTs. Continuous perfusion generated by placing the chips on a rocker resulted in greater vascularization, improved anastomosis, and enhanced formation of hybrid vessels. Since mechanistic studies on molecular pathways mediating anastomosis are challenging *in vivo*, this platform could provide an alternative way to understand this process *in vitro*. Furthermore, these networks developed robust lumens that contracted rhythmically in synchrony with surrounding CMs. This rhythmic contraction of CMs resulted in the generation of bidirectional flow as indicated by oscillatory movement of fluorescent beads into and through the organized vascular network inside of the MT. Currently, no animal models are available that model ischemic heart disease caused by coronary

Figure 2. Mechanism of the intra-microvascular network formation

(A) Schematic of the experimental setup. MTs were generated combining hiPSC-CMs, hiPSC-ECs (green, GFP), and hiPSC-CFs. These MTs were cocultured with hiPSC-ECs (red, mCherry) and HBVPs in chips.

(B and C) Representative images of outside-in (B) and inside-out (C) anastomosis and formation of hybrid vessels by interconnection of internal microvascular network (green, GFP) and external vascular network (red, mCherry) (20×). Scale bar, 150 μm.

(D and E) Representative confocal images of hybrid vessels visible in (B) and (C), respectively. Internal hiPSC-ECs (green, GFP) and external hiPSC-ECs (orange, mCherry). Images displaying maximum projection in xyz (i), xy (ii), and yz cross-sectional perspectives (iii) (40×). Scale bar, 100 μm. Arrows indicate the anastomosed points and hybrid lumens.

(F) Quantification of number of vascularized MTs (%, number vascularized MTs/total number of MTs). MTs contained CMs from three different hiPSC lines. Error bars are shown as mean ± SD from N = 3; three independent experiments with at least 10 MTs in each experiment. One-way ANOVA; ns, not significant.

(G) Quantification of number of anastomosed MTs (%, #anastomosed MTs/total # of MTs) in independent experiments. Error bars are shown as mean ± SD from N = 6; six independent experiments with at least nine MTs in each experiment. Student's t test, $*p < 0.05$.

See also Figure S2.

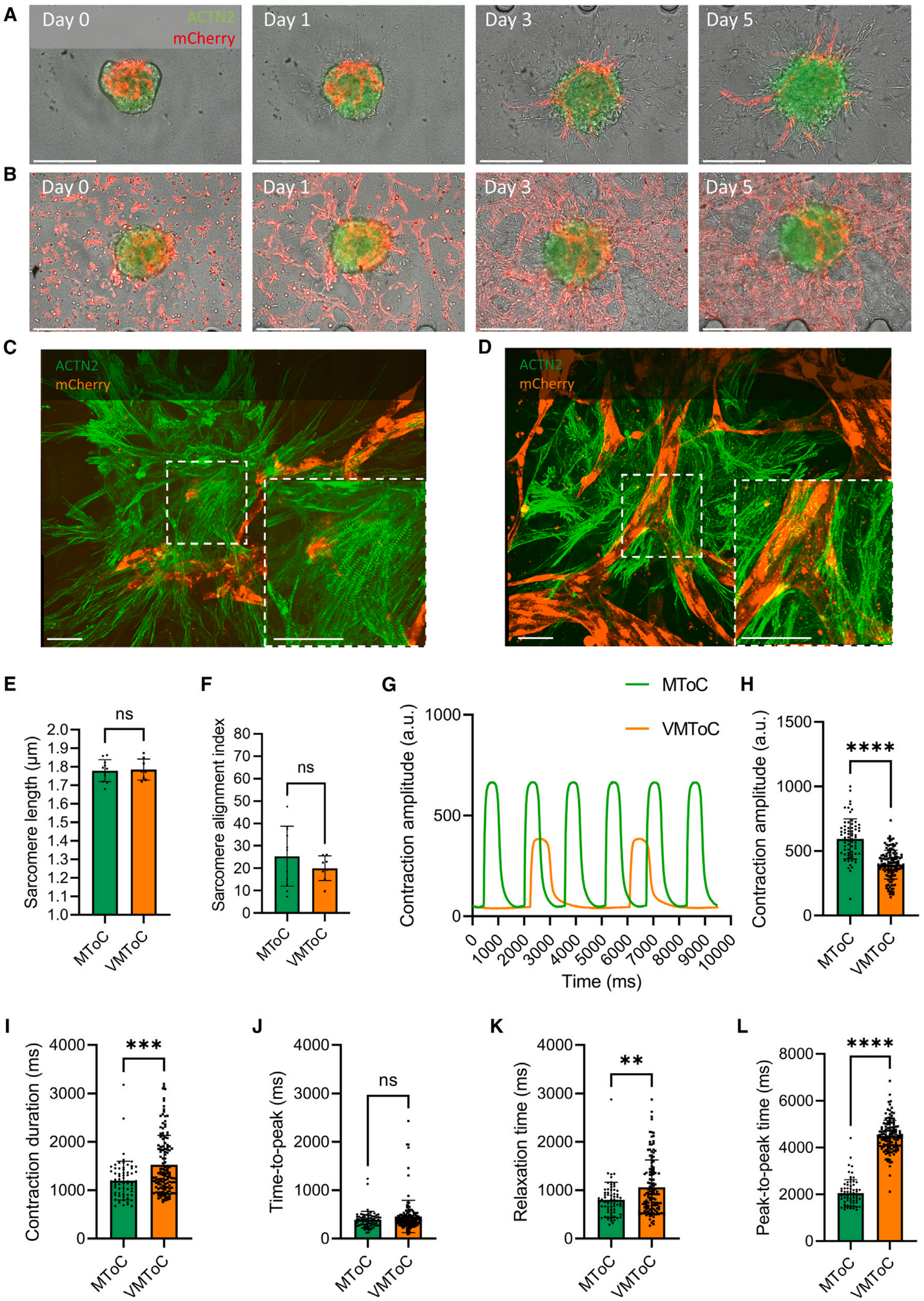

(legend on next page)

microvascular obstruction (Niccoli et al., 2016; Sorop et al., 2020). Therefore, vascularized and perfusable cardiac MTs could be useful in studying mechanisms underlying microvascular obstruction using cardiac and vascular cells derived from patients.

Previously we demonstrated that including ECs and cardiac fibroblasts (CFs) in MTs enhances CM maturation (Giacomelli et al., 2020). However, no differences in sarcomere organization were found between VMToC and MToC, indicating that further enhancement was not induced by the external vascular network. VMToCs showed characteristic low beat rates, but nevertheless, they continued to contract rhythmically throughout the experiments. Analysis of VMToC contractile parameters showed increased contraction duration and relaxation times. Notably, the increase in contraction duration in VMToC was not rate dependent and was maintained upon pacing at 0.8 and 1 Hz.

We further demonstrated that EC-CM crosstalk could be captured in VMToC upon delivery of stimuli via the external vascular network. Inhibition of NOS using L-NAME in VMToCs reversed the slow beating and decreased contraction duration, relaxation, and peak-to-peak time. At the same time, L-NAME had no significant effect in MToC after 1- or 6-h incubation, except for an increase in peak-to-peak time. The effect of L-NAME in VMToC could be explained by a possible increase in endothelium-derived endothelin (ET-1) production upon NOS inhibition (Czóbel et al., 2009; Kourembanas et al., 1993). Since VMToCs contain more ECs than MToCs, this could result in a pronounced response to NOS inhibition that was not observed in MToCs. Similarly, stimulation with IL-1β for 12 h resulted in upregulation of several pro-inflammatory cytokines, such as IL-6, IL-8, and MCP-1 in VMToC, but not in MToC, suggesting that only the vascular units were effectively responding to pro-inflammatory cytokine stimulation that are known contributors to EC dysfunction and heart failure. Stimulation with IL-1β decreased contraction duration, relaxation time, and peak-to-peak time. This was somewhat unexpected since IL-1β and IL-6 are known as negative inotropes that increase NO levels via upregulating the expression of inducible NOS (iNOS) in isolated CMs (Segers et al., 2018). On the other hand, IL-1β and IL-6 decrease endothelial NOS (eNOS) expression and activity in ECs (Hung et al., 2010; Kawasaki et al., 2015; Saura et al., 2006). Although we have not examined expression of iNOS and eNOS upon IL-1β treatment in VMToC vs. MToC, the fact that the IL-1β inhibitory effect was only observed in VMToC indicates the importance of the vascular component in mediating the response in CMs.

Finally, although the formation of the vascular networks was robust in VMToC, we observed some batch-to-batch and plate-to-plate variability in the degree of vascularization. Vascularization of MTs was mostly through the anastomosis, so the extent of pre-vascularization in MTs might contribute to variability. In some cases where MTs were not well-vascularized, vascularization was improved by introducing continuous perfusion during later stages of culture (data not shown). In addition, inter-batch and inter-line variability might be the result of differences in basal contractile parameters and/or drug responses in VMToCs. We showed consistency between two different hiPSC lines, but further validation using more hiPSC lines may be of value as NOS levels may differ between CMs and ECs from different lines and thus affect contractile dynamics and drug responses to different extents.

In summary, we demonstrated that 3D cardiac MTs can be integrated into microfluidic chips with the external vascular network formed by hiPSC-ECs and HBVPs. Using this model, we demonstrated that cardiac MTs can be efficiently vascularized within days, and the lumenized vascular networks in and around the MTs can be perfused. This may ultimately allow selective nutrient or drug delivery to the cells via the endothelial barrier inside the MTs, mimicking heart tissue *in vivo* even more closely. Our vascularized cardiac MT model thus provides a foundation for studies on organ-specific cellular communication, specifically for the endothelial barrier, drug screening, and disease modeling.

Figure 3. Characterization of contractile dynamics of cardiac MTs in MToC and VMToC

(A and B) Representative images of MTs in chips on days 0, 1, 3, and 5 in MToC (A) and VMToC (B) (10×). Scale bar, 300 μm.

(C and D) Representative confocal images of sarcomeres showing hiPSC-ECs (orange, mCherry) and hiPSC-CMs (green, ACTN2) in MToC (C) and VMToC (D) (40×). White dashed box is the area that is zoomed 100×. Scale bar, 50 μm.

(E and F) Quantification of the sarcomere parameters: sarcomere length (E); sarcomere alignment index (F); error bars are shown as mean ± SD from MToC N = 3, n = 11; VMToC N = 4, n = 8; three or four independent experiments with at least eight MTs.

(G) Representative beating traces of MTs from MToC (green trace) and VMToC (orange trace).

(H–L) Quantification of the contraction parameters: contraction amplitude (H), contraction duration (I), time to peak (J), relaxation time (K), and peak-to-peak time (L) in MTs with AICS-0075 hiPSC-CMs. Error bars are shown as mean ± SD from MToC N = 3, n = 69; VMToC N = 3, n = 132; three independent experiments with 16 or 24 MTs from at least six different microfluidic channels each experiment. Student's t test (E), Wilcoxon-Mann-Whitney test (F and H–L). ***$p < 0.001$, ****$p < 0.0001$; ns, not significant.

See also Figure S3.

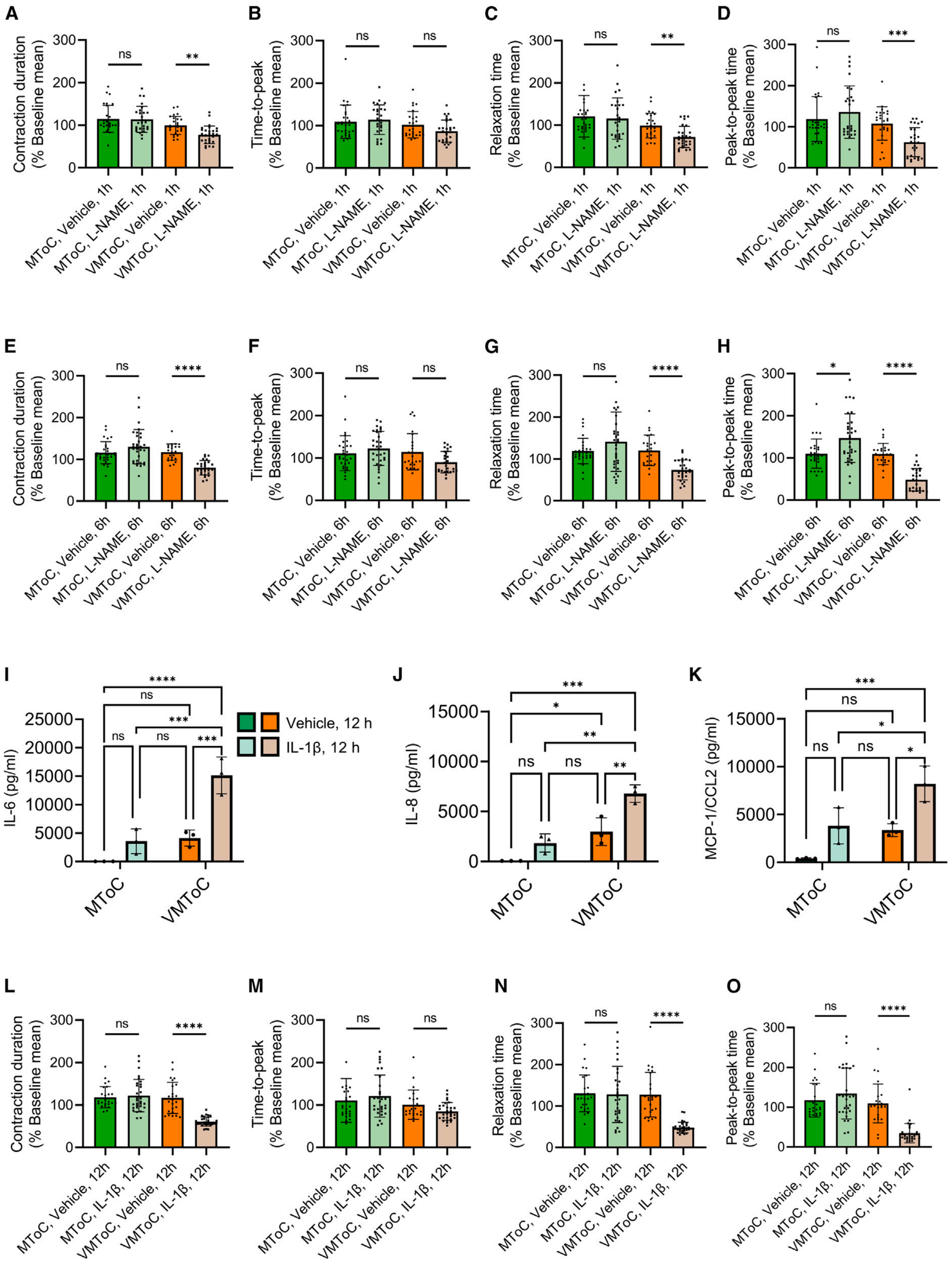

(legend on next page)

EXPERIMENTAL PROCEDURES

Resource availability

Corresponding author

Requests for further information or more detailed protocols should be directed to and will be fulfilled by the corresponding author, Valeria V. Orlova (v.orlova@lumc.nl).

Materials availability

This study did not generate new unique reagents.

Data and code availability

Data will be shared with the research community upon request. No code or standardized datasets were generated.

hiPSC lines

The medical ethical committee in Leiden University, the Netherlands, approved the use of hiPSCs in this study. A detailed list of the hiPSC lines and batches used for each experiment can be found in Table S1.

3D cardiac microtissue formation

3D cardiac MTs were formed using hiPSC-CMs, hiPSC-ECs, and hiPSC-CFs following a previously established protocol (Giacomelli et al., 2020).

Microfluidic chip culture

Cell preparation prior to chip seeding is described in the supplemental information. Commercially available microfluidic chips (AIM Biotech) were used. Cell and MT mixtures were prepared as follows: (1) four MTs/channel in combination with 25 × 10^6 hiPSC-ECs/mL and 5 × 10^6 HBVP cells/mL (5:1 ratio) (VMToC); (2) 25 × 10^6 hiPSC-ECs/mL and 5 × 10^6 HBVP cells/mL (VoC) or (3) four MTs/channel (MToC) were resuspended in endothelial growth medium-2 (EGM-2, Lonza) supplemented with thrombin (4 U/mL) and then gently mixed with fibrinogen (final concentration 3 mg/mL, Sigma) at 1:1 vol ratio. Cell/hydrogel mixture was quickly loaded into the middle gel-loading channel of the microfluidic chip. Chips were incubated at room temperature for 15 min. To support vascular network formation in the presence of cardiac MTs, we used a mixture of CM and EC growth medium (bovine serum albumin and essential lipids medium and EGM-2, 50:50), supplemented with vascular endothelial growth factor (50 ng/mL). The γ-secretase inhibitor DAPT (10 μM) was also added to the medium on day 1 for 24 h. Intermittent gravity-driven flow in the whole chamber was induced by creating hydrostatic pressure through the addition of 100 μL medium to the right media ports and 50 μL media to left media ports in the medium channel. Medium was refreshed daily. For the continuous perfusion experiments, microfluidic chips were placed on the interval rocker platform (Perfusion rocker, MIMETAS) set at a 5° inclination angle and an 8-min interval from day 0 onward. Chips were maintained until day 7 and characterized between days 5 and 7.

Statistical analysis

Statistical analysis was performed using GraphPad Prism 9. Student's t test or one-way or two-way ANOVA for paired or unpaired measurements was applied as appropriate to test for differences in means between groups/conditions. Kruskal-Wallis test and Wilcoxon-Mann-Whitney test were used when the normality assumption did not hold. Data are expressed and plotted as the mean ± SD as indicated in figure legend. Detailed statistics and exact p values are indicated in each figure legend. Statistical significance was defined as $p < 0.05$.

SUPPLEMENTAL INFORMATION

Supplemental information can be found online at https://doi.org/10.1016/j.stemcr.2023.06.001.

AUTHOR CONTRIBUTIONS

Conceptualization, V.V.O.; methodology, U.A. and V.V.O.; software, U.A., D.N., J.S., and B.v.M.; validation, U.A., M.B., and V.M.; formal analysis, U.A.; investigation, U.A., M.B., V.M., D.M.N., R.W.J.v.H., J.M.S., F.E.v.d.H., B.v.M., and M.V.C.; visualization, U.A.; resources, C.L.M. and V.V.O.; writing – original draft,

Figure 4. Altered contractile dynamics and inflammatory response of cardiac MTs regulated by EC-CM communication in VMToC but not in MToC

(A–H) Quantification of the contraction parameters presented as percentage change from the baseline mean of spontaneous beating condition, after 1 h (A–D) and 6 h (E–H) incubation with vehicle or L-NAME (1 mM): contraction duration (A and E); time to peak (B and F); relaxation time (C and G); peak-to-peak time (D and H) in MTs with LUMC0059iCTRL03 hiPSC-CMs. Error bars are shown as mean ± SD from MToC N = 3, n > 28 (vehicle) and n > 27 (L-NAME); VMToC N = 3, n > 24 (vehicle) and n > 27 (L-NAME); three independent experiments with at least seven MTs in each experiment.

(I–K) Quantification of pro-inflammatory cytokines from the medium after 12 h incubation of IL-1β (10 ng/mL): IL-6 (I); IL-8 (J); MCP1/CCL2 (K) in MTs with LUMC0059iCTRL03 hiPSC-CMs. Y axis shows concentration (pg/mL). Error bars are shown as mean ± SD. MToC N = 3, n > 3; VMToC N = 3, n > 3; three independent experiments; medium was collected from at least with three different microfluidic channels in each experiment.

(L–O) Quantification of the contraction parameters presented as percentage change from the baseline mean of spontaneous beating condition after 12 h incubation with vehicle or IL-1β (10 ng/mL): contraction duration (L); time to peak (M); relaxation time (N); peak-to-peak time (O) in MTs with LUMC0059iCTRL03 hiPSC-CMs. Error bars are shown as mean ± SD from MToC N = 3, n = 26 (vehicle) and n = 30 (IL-1β); VMToC N = 3, n = 25 (vehicle) and n = 31 (IL-1β); three independent experiments with at least seven MTs in each experiment. Kruskal-Wallis test with Dunn's multiple comparisons test (A–H, L–O), two-way ANOVA with Šidák's multiple comparisons test (I–K), *$p < 0.05$, **$p < 0.01$, ***$p < 0.001$; ****$p < 0.0001$; ns, not significant.

See also Figure S4.

U.A., M.B., C.L.M., and V.V.O.; writing – review & editing, U.A., C.L.M., and V.V.O.; supervision, C.L.M., and V.V.O.; project administration, V.V.O.; funding acquisition, C.L.M. and V.V.O.

ACKNOWLEDGMENTS

We acknowledge LUMC confocal imaging facility for help with imaging and the Allen Cell Collection, available from Coriell Institute for Medical Research, for providing materials. We thank Mervyn Mol for help with CM differentiation. Images were generated using Biorender.com.

This work was supported by the European Union's Horizon 2020 research and innovation program under grant agreement No. 812954; the Netherlands Organ-on-Chip Initiative, which is an NWO Gravitation project (024.003.001) funded by the Ministry of Education, Culture and Science of the government of the Netherlands; the LymphChip project with project number NWA-ORC 2019 1292.19.019 of the NWA research program "Research on Routes by Consortia (ORC)," which is funded by the Netherlands Organisation for Scientific Research (NWO); and The Novo Nordisk Foundation Center for Stem Cell Medicine that is supported by a Novo Nordisk Foundation grant (NNF21CC0073729).

CONFLICT OF INTERESTS

C.L.M. is co-founder of Ncardia bv.

Received: September 14, 2022
Revised: June 2, 2023
Accepted: June 2, 2023
Published: June 29, 2023

REFERENCES

Abe, Y., Watanabe, M., Chung, S., Kamm, R.D., Tanishita, K., and Sudo, R. (2019). Balance of interstitial flow magnitude and vascular endothelial growth factor concentration modulates three-dimensional microvascular network formation. APL Bioeng. *3*, 036102.

Amersfoort, J., Eelen, G., and Carmeliet, P. (2022). Immunomodulation by endothelial cells — partnering up with the immune system? Nat. Rev. Immunol. *22*, 576–588.

Arslan, U., Moruzzi, A., Nowacka, J., Mummery, C.L., Eckardt, D., Loskill, P., and Orlova, V.V. (2022). Microphysiological stem cell models of the human heart. Mater. Today. Bio *14*, 100259.

Berry, C., and Duncker, D.J. (2020). Coronary microvascular disease: the next Frontier for Cardiovascular Research. Cardiovasc. Res. *116*, 737–740.

Campostrini, G., Meraviglia, V., Giacomelli, E., Helden, R.W.J., van, Yiangou, L., Davis, R.P., Bellin, M., Orlova, V.V., and Mummery, C.L. (2021). Generation, functional analysis and applications of isogenic three-dimensional self-aggregating cardiac microtissues from human pluripotent stem cells. Nat Protoc *16*, 2213–2256. https://doi.org/10.1038/s41596-021-00497-2.

Czóbel, M., Kaszaki, J., Molnár, G., Nagy, S., and Boros, M. (2009). Nonspecific inhibition of nitric oxide synthesis evokes endothelin-dependent increases in myocardial contractility. Nitric Oxide *21*, 201–209.

Galie, P.A., Nguyen, D.H.T., Choi, C.K., Cohen, D.M., Janmey, P.A., and Chen, C.S. (2014). Fluid shear stress threshold regulates angiogenic sprouting. Proc. Natl. Acad. Sci. USA *111*, 7968–7973.

Giacomelli, E., Meraviglia, V., Campostrini, G., Cochrane, A., Cao, X., van Helden, R.W.J., Krotenberg Garcia, A., Mircea, M., Kostidis, S., Davis, R.P., et al. (2020). Human-iPSC-Derived cardiac stromal cells enhance maturation in 3D cardiac microtissues and reveal non-cardiomyocyte contributions to heart disease. Cell Stem Cell *26*, 862–879.e11.

Hung, M.J., Cherng, W.J., Hung, M.Y., Wu, H.T., and Pang, J.H.S. (2010). Interleukin-6 inhibits endothelial nitric oxide synthase activation and increases endothelial nitric oxide synthase binding to stabilized caveolin-1 in human vascular endothelial cells. J. Hypertens. *28*, 940–951.

Kawasaki, Y., Yokobayashi, E., Sakamoto, K., Tenma, E., Takaki, H., Chiba, Y., Otashiro, T., Ishihara, M., Yonezawa, S., Sugiyama, A., and Natori, Y. (2015). Angiostatin prevents IL-1β-induced down-regulation of eNOS expression by inhibiting the NF-κB cascade. J. Pharmacol. Sci. *129*, 200–204.

Kourembanas, S., McQuillan, L.P., Leung, G.K., and Faller, D.V. (1993). Nitric oxide regulates the expression of vasoconstrictors and growth factors by vascular endothelium under both normoxia and hypoxia. J. Clin. Invest. *92*, 99–104.

Mansour, A.A., Gonçalves, J.T., Bloyd, C.W., Li, H., Fernandes, S., Quang, D., Johnston, S., Parylak, S.L., Jin, X., and Gage, F.H. (2018). An in vivo model of functional and vascularized human brain organoids. Nat. Biotechnol. *36*, 432–441.

Niccoli, G., Scalone, G., Lerman, A., and Crea, F. (2016). Coronary microvascular obstruction in acute myocardial infarction. Eur. Heart J. *37*, 1024–1033.

Ryan, A.R., England, A.R., Chaney, C.P., Cowdin, M.A., Hiltabidle, M., Daniel, E., Gupta, A.K., Oxburgh, L., Carroll, T.J., and Cleaver, O. (2021). Vascular deficiencies in renal organoids and ex vivo kidney organogenesis. Dev. Biol. *477*, 98–116.

Sala, L., van Meer, B.J., Tertoolen, L.G.J., Bakkers, J., Bellin, M., Davis, R.P., Denning, C., Dieben, M.A.E., Eschenhagen, T., Giacomelli, E., et al. (2018). Musclemotion: a versatile open software tool to quantify cardiomyocyte and cardiac muscle contraction in vitro and in vivo. Circ. Res. *122*, e5–e16.

Saura, M., Zaragoza, C., Bao, C., Herranz, B., Rodriguez-Puyol, M., and Lowenstein, C.J. (2006). Stat3 mediates interelukin-6 inhibition of human endothelial nitric-oxide synthase expression. J. Biol. Chem. *281*, 30057–30062.

Segers, V.F.M., Brutsaert, D.L., and De Keulenaer, G.W. (2018). Cardiac remodeling: endothelial cells have more to say than just NO. Front. Physiol. *9*, 382.

Sorop, O., Van De Wouw, J., Chandler, S., Ohanyan, V., Tune, J.D., Chilian, W.M., Merkus, D., Bender, S.B., and Duncker, D.J. (2020). Experimental animal models of coronary microvascular dysfunction. Cardiovasc. Res. *116*, 756–770.

Stein, J.M., Arslan, U., Franken, M., De Greef, J.C., E Harding, S., Mohammadi, N., Orlova, V.V., Bellin, M., Mummery, C.L.,

and Van Meer, B.J. (2022). Software tool for automatic quantification of sarcomere length and organization in fixed and live 2D and 3D muscle cell cultures in vitro. Curr. Protoc. *2*, e462.

Takebe, T., Sekine, K., Enomura, M., Koike, H., Kimura, M., Ogaeri, T., Zhang, R.R., Ueno, Y., Zheng, Y.W., Koike, N., et al. (2013). Vascularized and functional human liver from an iPSC-derived organ bud transplant. Nature *499*, 481–484.

Zhang, S., Wan, Z., and Kamm, R.D. (2021). Vascularized organoids on a chip: strategies for engineering organoids with functional vasculature. Lab Chip *21*, 473–488.

Zhang, S., Wan, Z., Pavlou, G., Zhong, A.X., Xu, L., Kamm, R.D., Zhang, S., Wan, Z., Pavlou, G., Zhong, A.X., et al. (2022). Interstitial flow promotes the formation of functional microvascular networks in vitro through upregulation of matrix metalloproteinase-2. Adv. Fun *32*, 2206767.

Stem Cell Reports, Volume *18*

Supplemental Information

Vascularized hiPSC-derived 3D cardiac microtissue on chip

Ulgu Arslan, Marcella Brescia, Viviana Meraviglia, Dennis M. Nahon, Ruben W.J. van Helden, Jeroen M. Stein, Francijna E. van den Hil, Berend J. van Meer, Marc Vila Cuenca, Christine L. Mummery, and Valeria V. Orlova

Inventory of Supplemental Information

Supplemental Figures and Legends:

Figure S1. Cardiac MTs induce pulsative intra-microvascular flow. Related to Figure 1.
Figure S2. Continuous perfusion enhances vascularization of cardiac MTs. Related to Figure 2.
Figure S3. Characterization of contraction parameters upon pacing. Related to Figure 3.
Figure S4. Altered contractile dynamics of cardiac MTs upon inhibition of EC-CM communication in VMToC but not in MToC. Related to Figure 4.

Supplemental Tables:

Supplemental Table 1. List of hiPSC lines and cell batches used per experiment.

Supplemental Videos:

Supplemental Video 1. 3D reconstruction of VMToC on the left showing lumenized microvascular network formed by hiPSC-ECs (orange, mCherry) embedded in hiPSC-CMs (green, ACTN2). 3D reconstruction of MToC on the right showing that hiPSC-ECs (orange, mCherry) migrated out of the MTs and hiPSC-CMs (green, ACTN2) developed long protrusions. Related to Figure 1 and 3.

Supplemental Video 2. Simultaneous video recording of two channels for contraction (on the left) and perfusion of fluorescent beads (on the right) showing hiPSC-CMs (green, ACTN2) and hiPSC-ECs (orange, mCherry). (20x). Related to Figure 1.

Supplemental Video 3. Simultaneous video recording of two channels for contraction (on the left) and perfusion of fluorescent beads (on the right) showing hiPSC-CMs (green, ACTN2) and hiPSC-ECs (orange, mCherry). (40x). Related to Figure 1.

Supplemental Experimental Procedures

Supplemental References

Supplemental Figures and Legends

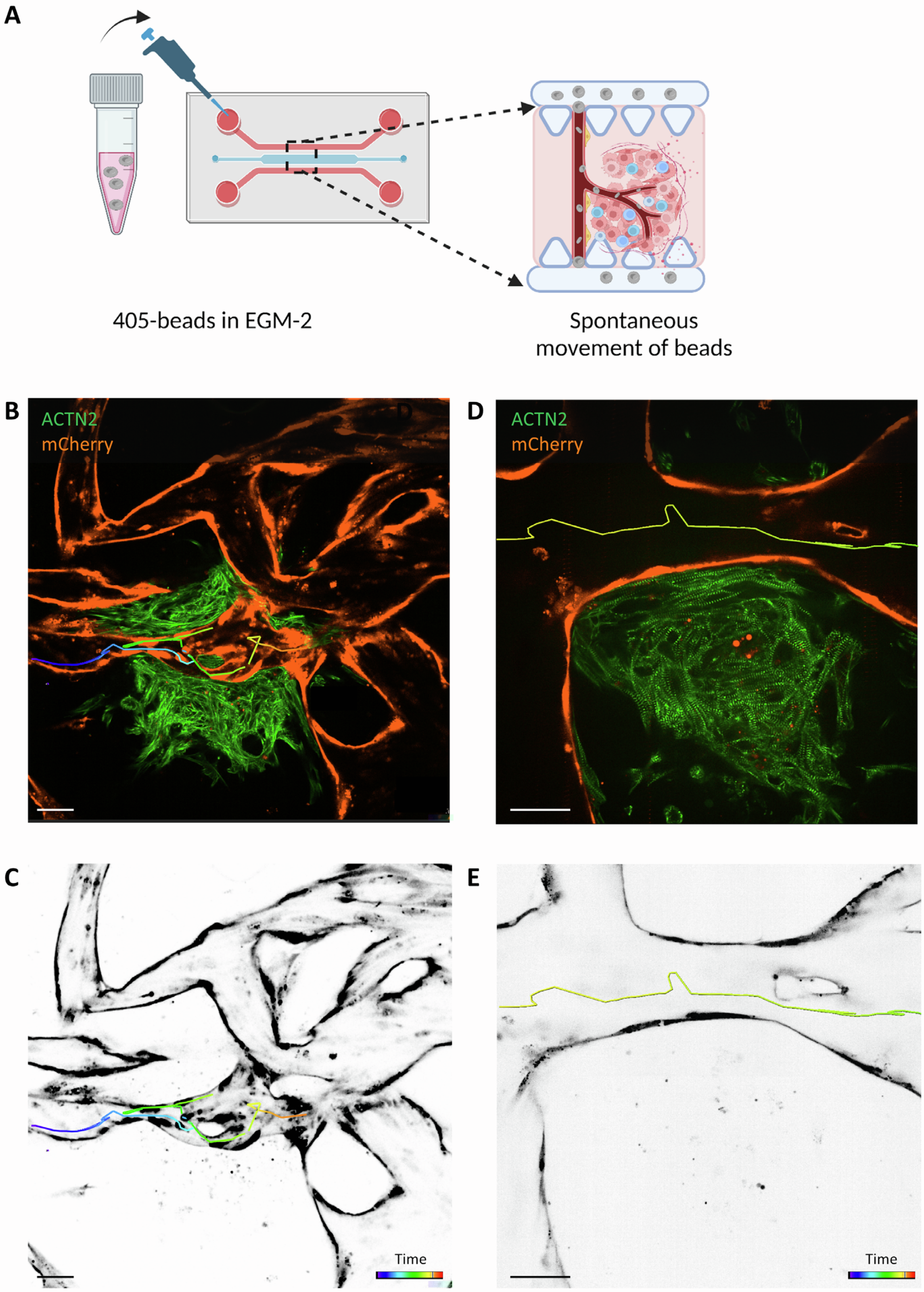

Figure S1. Cardiac MTs induce pulsative intra-microvascular flow. Related to Figure 1. (A) Schematic of the fluorescent beads (2 μm) perfusion experiment. Please see Supplemental Experimental Setup for experimental details. (B, D) Representative confocal images showing hiPSC-ECs (orange, mCherry) hiPSC-CM (green, ACTN2) and perfusion of 2 μm fluorescent beads (traces) in VMToC. (B is 20x and D is 40x with Scale bar, 50 μm. (C, E) Representative confocal images in black and white showing only vascular networks (black) and traces of beads inside (colored traces). Color coded bars represent time. Images are duplications of B and D. Scale bar, 50 μm.

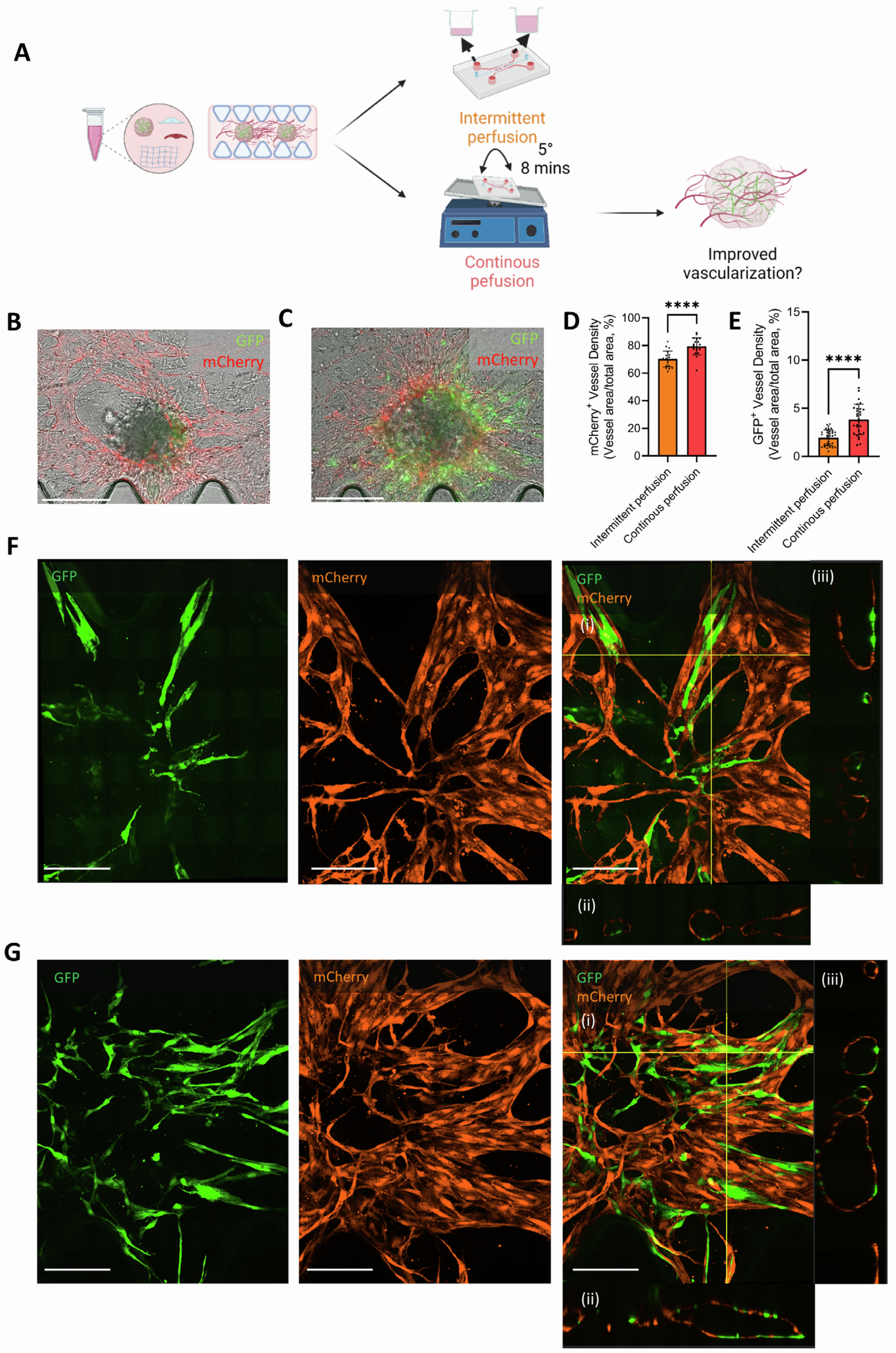
A
Intermittent perfusion
5°
8 mins
Continous pefusion
Improved vascularization?
B
GFP
mCherry
C
GFP
mCherry
D
****
mCherry+ Vessel Density (Vessel area/total area, %)
100
80
60
40
20
0
Intermittent perfusion
Continous perfusion
E
****
GFP+ Vessel Density (Vessel area/total area, %)
15
10
5
0
Intermittent perfusion
Continous perfusion
F
GFP
mCherry
GFP
mCherry
(i)
(ii)
(iii)
G
GFP
mCherry
GFP
mCherry
(i)
(ii)
(iii)

Figure S2. Continuous perfusion enhances vascularization of cardiac MTs. Related to Figure 2. (A) Schematic of the experimental setup for intermittent and continuous perfusion conditions. Chips were kept under intermittent gravity-driven flow with the medium exchange every 24 h or placed on the rocker (at a 5 degrees inclination angle and 8 min interval) to induce continuous perfusion through passive leveling between the reservoirs. (B, C) Representative images of VMToC under intermittent perfusion (B) and continuous perfusion (C) where anastomosis is visible by the formation of hybrid vessels (10x). Scale bar, 300 μm. (D) Quantification of $mCherry^+$ vessel density (%) in intermittent and continuous perfusion conditions. Error bars are shown as ±SD from N = 3, n = 19 (intermittent perfusion) and n = 20 (continuous perfusion); three independent experiments with at least six microfluidic channels per experiment. (E) Quantification of GFP^+ vessel density (%) in intermittent and continuous perfusion conditions. Error bars are shown as ±SD from N = 3, n = 35 (intermittent perfusion) and n = 29 (continuous perfusion); three independent experiments with at least six microfluidic channels per experiment. (F, G) Representative confocal images of hybrid vessels formed in intermittent perfusion (F) or continues perfusion (G) conditions. Internal hiPSC-EC (green, GFP), external hiPSC-EC (orange, mCherry). Images displaying xyz (i), xy (ii), and yz cross-sectional perspectives (iii) (40×). Scale bar, 150 μm.
Wilcoxon-Mann-Whitney test (D, E), $****p < 0.0001$.

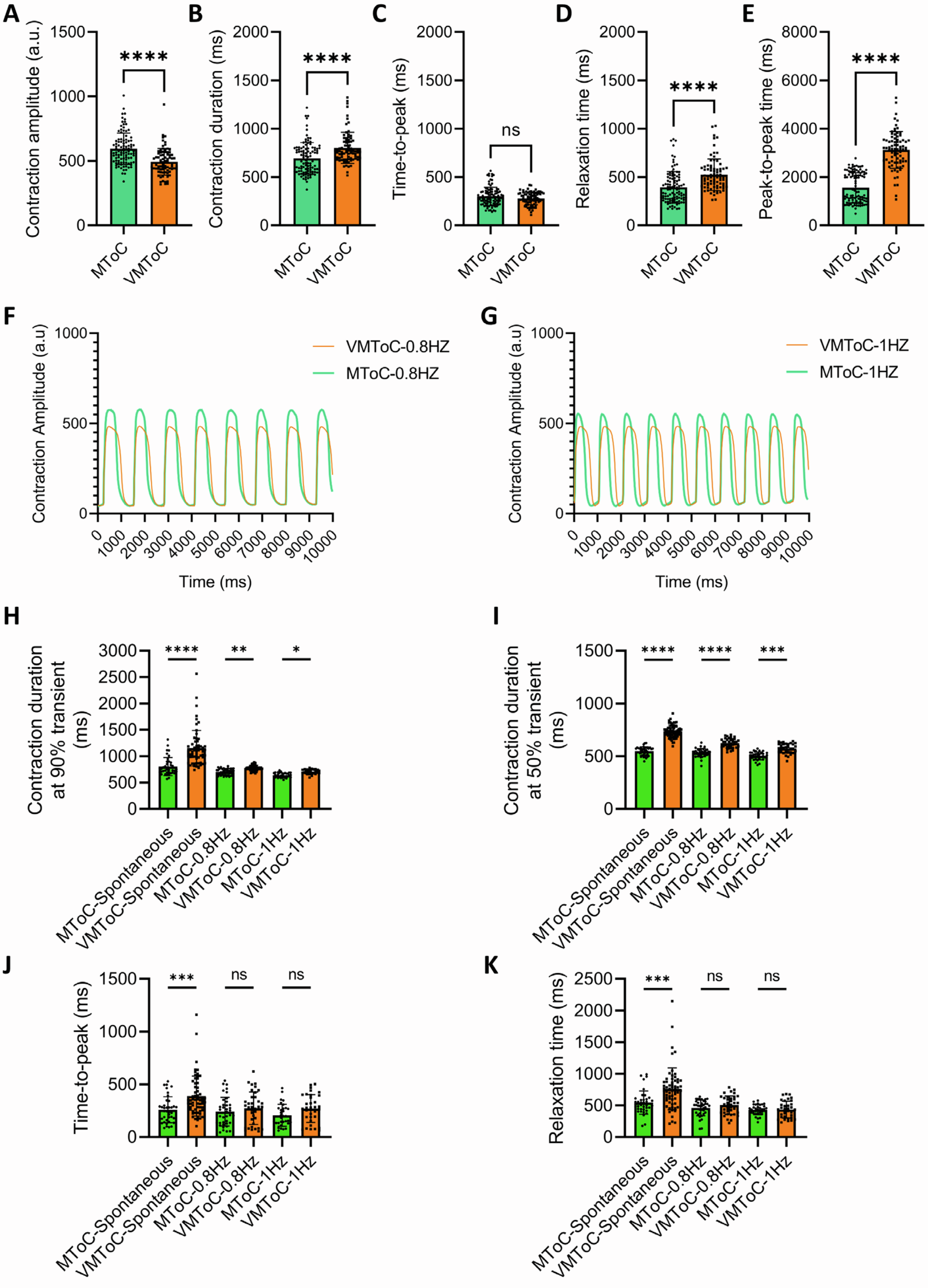
A
B
C
D
E
Contraction amplitude (a.u.)
Contraction duration (ms)
Time-to-peak (ms)
Relaxation time (ms)
Peak-to-peak time (ms)
****
ns
MToC
VMToC
F
G
Contraction Amplitude (a.u)
VMToC-0.8HZ
MToC-0.8HZ
VMToC-1HZ
MToC-1HZ
Time (ms)
H
I
Contraction duration at 90% transient (ms)
Contraction duration at 50% transient (ms)
MToC-Spontaneous
VMToC-Spontaneous
MToC-0.8Hz
VMToC-0.8Hz
MToC-1Hz
VMToC-1Hz
J
K
Time-to-peak (ms)
Relaxation time (ms)

Figure S3. Characterization of contraction parameters upon pacing. Related to Figure 3. (A-E) Quantification of the contraction parameters of MToC and VMToC: contraction amplitude (A); contraction duration (B); time-to-peak (C); relaxation time (D); peak-to-peak time (E) in MTs with LUMC0059iCTRL03 hiPSC-CMs. Error bars are shown as ±SD from MToC N = 3, n>30; VMToC N =3, n>26; three independent experiments with 26 MTs from at least six different microfluidic channels in each experiment. (F-G) Representative beating traces at 0.8 Hz (A) and 1 Hz (B). (H-K) Quantification of the contraction parameters during spontaneous beating, at 0.8 Hz and 1 Hz paced MTs: contraction duration at 90% transient (H); contraction duration at 50% transient (I); time-to-peak (J); relaxation time (K) in MTs with AICS-0075 hiPSC-CMs. Error bars are shown as ±SD from MToC N = 3, n >10; VMToC N =3, n>10; three independent experiments with 10 MTs from at least three different microfluidic channels were carried out for each experiment.
Wilcoxon-Mann-Whitney test (A-E), Kruskal-Wallis test with Dunn's multiple comparisons test (H-K); ∗p < 0.05, ∗∗p < 0.01; ∗∗∗p < 0.001, ∗∗∗∗p < 0.0001; ns, not significant.

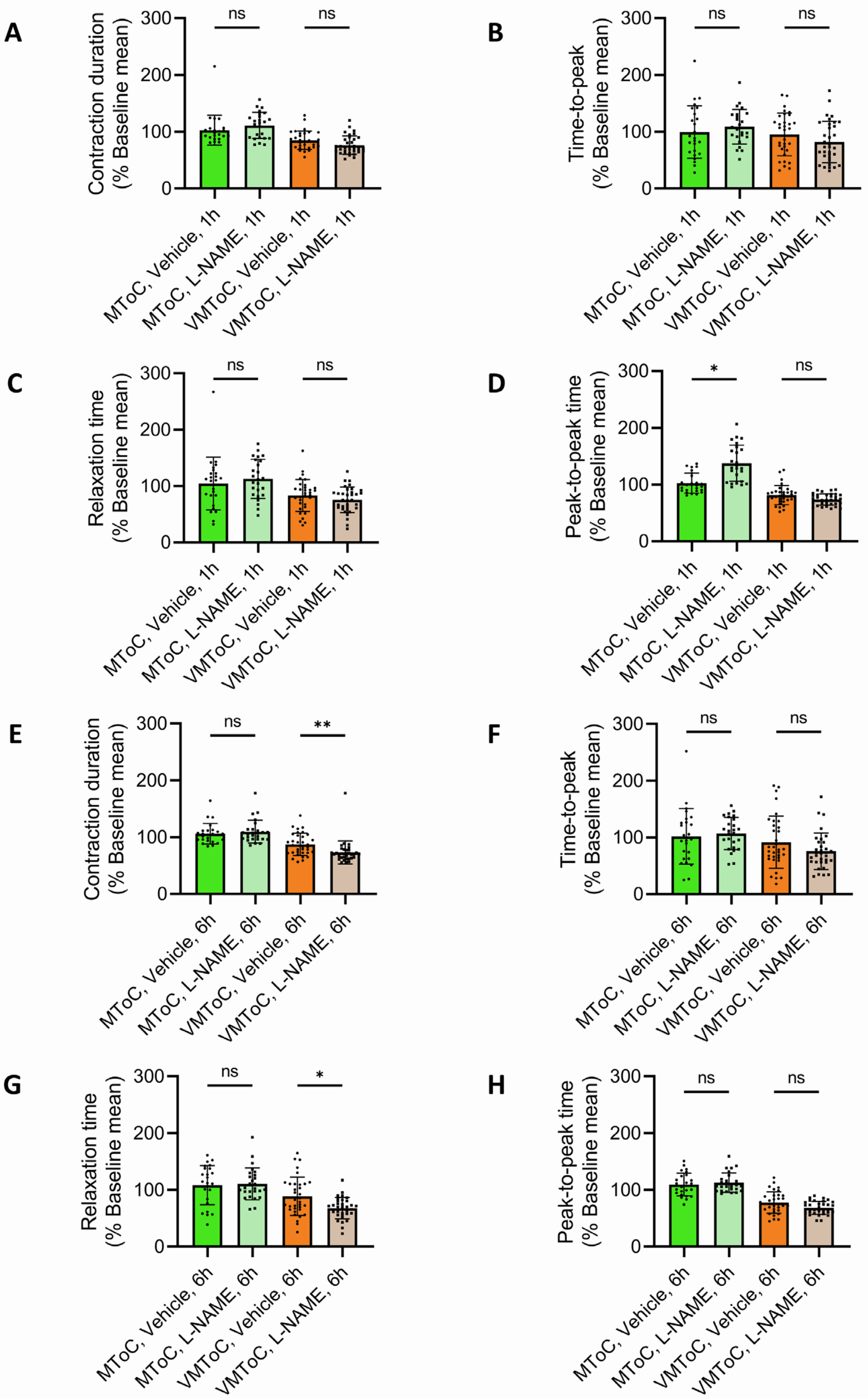
A
B
C
D
E
F
G
H
Contraction duration (% Baseline mean)
Time-to-peak (% Baseline mean)
Relaxation time (% Baseline mean)
Peak-to-peak time (% Baseline mean)
ns
*
**
MToC, Vehicle, 1h
MToC, L-NAME, 1h
VMToC, Vehicle, 1h
VMToC, L-NAME, 1h
MToC, Vehicle, 6h
MToC, L-NAME, 6h
VMToC, Vehicle, 6h
VMToC, L-NAME, 6h

Figure S4: Altered contractile dynamics of cardiac MTs upon inhibition of EC-CM communication in VMToC but not in MToC. Related to Figure 4. (A-H) Quantification of the contraction parameters presented as percentage change from the baseline mean of spontaneous beating condition, after 1 h (A-D) and 6 h (E-H) incubation of L-NAME (1 mM): contraction duration (A, E); time-to-peak (B, F); relaxation time (C, G); peak-to-peak time (D, H) in MTs with AICS-0075 hiPSC-CMs. Error bars are shown as ±SD from MToC N = 3, n >6; VMToC N =3, n > 7; three independent experiments with at least 6 MTs from at least four different microfluidic channels in each experiment. Data is normalized to baseline mean of each set for vehicle and L-NAME group of MTs.
Kruskal-Wallis test with Dunn’s multiple comparisons test (A-H). $*p < 0.05$, $**p < 0.01$; ns, not significant.

Supplemental Tables:

Table S1. List of hiPSC lines and cell batches used in this study.

**Figures**	**hiPSC-CM**		**hiPCS-EC (internal vascular network)**		**hiPSC-EC (external vascular network)**		**hiPSC-CF**	
	**Cell Line**	**Number of batches**	**Cell Line**	**Number of batches**	**Cell Line**	**Number of batches**	**Cell Line**	**Number of batches**
Figure 1, 3, S1, S3F-K, S4, Video S1, S2, S3	AICS-0075	3	NCRM-1 (mCherry)	3	NCRM-1 (mCherry)	4	LUMC0020 iCTRL06	3
Figure 2B-E, 2G, 2F (Line 2), S2	LUMC0020 iCTRL06	3	NCRM-1 (GFP)	1	NCRM-1 (mCherry)	4	LUMC0020 iCTRL06	3
Figure 2F	AICS-0075 (Line 1); LUMC0020 iCTRL06 (Line 2); LUMC0059 iCTRL03 (Line 3)	1<br>1<br>1	NCRM-1 (mCherry)	1	NCRM-1 (mCherry)	1	LUMC0020 iCTRL06	1
Figure 2F (line 3), 4, S3A-E	LUMC0059 iCTRL03	1	NCRM-1 (mCherry)	1	NCRM-1 (mCherry)	1	LUMC0020 iCTRL06	1

Supplemental Experimental Setup

hiPSC lines and maintenance

hiPSCs were maintained on recombinant vitronectin-coated plates in TeSR-E8, all from STEMCELL Technologies, according to the manufacturer's instructions. The following hiPSC lines were used in this study: LUMC0059iCTRL03 (generated from skin fibroblasts, https://hpscreg.eu/cell-line/LUMCi026-A), LUMC0020iCTRL06 (generated from skin fibroblasts, https://hpscreg.eu/cell-line/LUMCi028-A) (Zhang et al., 2014); NIH Center for Regenerative Medicine: NCRM-1 (generated from CD34+ cord blood cells, https://hpscreg.eu/cell-line/CRMi003- A), obtained from RUDCR Infinite Biologics at Rutgers University, was modified in-house with a mCherry or GFP expression cassette under the human cytomegalovirus (hCMV) early enhancer/chicken β actin (CAG) promoter using a previously established protocol (Rostovskaya et al., 2012); The Allen Cell Collection: AICS-0075 (generated from skin fibroblasts, https://hpscreg.eu/cell-line/UCSFi001-A-4) with mEGFP insertion site at ACTN2 was obtained from Coriell Institute for Medical Research. hiPSC lines and cell batches of hiPSC-CMs, hiPSC-ECs and hiPSC-CFs are listed in Table 1.

Differentiation of cardiomyocytes

CM differentiation of hiPSC was induced in monolayer as described previously (Berg et al., 2016; Giacomelli et al., 2017). Briefly, 25 × 10^3 cells per cm2 were seeded on plates coated with 75 µg/ml growth factor-reduced Matrigel (Corning) the day before the start of differentiation (day -1). On day 0, cardiac mesoderm was induced by changing E8 to B(P)EL medium (Bovine Serum Albumin [BSA] and Essential Lipids), supplemented with a cytokine mixture (20 ng/ml BMP4, R&D Systems; 20 ng/ml ACTIVIN A, Miltenyi Biotec; 1.5 µM GSK3 inhibitor CHIR99021, Axon Medchem). After 3 days, cytokines were removed and XAV939 which is WNT inhibitor (5 µM, Tocris) was added for 3 days. After this, B(P)EL medium was refreshed every 3 days.

Differentiation of cardiac fibroblasts

CFs were differentiated from hiPSC using an epicardial (EPI) monolayer differentiation protocol (Guadix et al., 2017) as a starting point (Giacomelli et al., 2020). Briefly, 25 × 10^3 cells per cm2 were seeded on Matrigel at day -1. On day 0, cardiac mesoderm was induced as described above. After 3 days, cytokines were removed and XAV939 (5 µM) was added for 3 days with BMP4 (30 ng/ml) and Retinoic Acid (RA; 1 µM; Sigma Aldrich). On day 6, B(P)EL medium supplemented with BMP4 (30 ng/ml) and RA (1 µM) was refreshed. On day 9, 15 × 10^3 per cm2 were seeded on plates coated with 5 µg/ml of fibronectin from bovine plasma (fibronectin; Sigma Aldrich) in B(P)EL medium supplemented with the TGFβ inhibitor SB431542 (10 µM; Tocris). By day 12, EPIs were confluent and ready for passaging or analysis. EPI cells (30 cm2 per vial) were cryopreserved in CryoStor CS10 medium (0.5 ml/vial; Stem Cell Technologies).

CF differentiation was induced in monolayer. Briefly, 25 × 10^3 EPIs were seeded per cm2 on tissue culture plates coated with vitronectin in B(P)EL medium supplemented with FGF2 (10 ng/ml; R&D Systems) on day 12. On day 13 and every 2 days thereafter, medium was refreshed with B(P)EL supplemented with FGF2 (10 ng/ml). After 6 days (on day 19), CFs were expanded by changing B(P)EL to Fibroblast Growth Medium 3 (FGM3; PromoCell). FGM3 was refreshed every 2 days for approximately 10 days in total. After 10 days (on day 29), CFs were confluent and ready to be passaged at 1:2 ratio. FGM3 was refreshed the day after passaging and every 2 days thereafter. CFs (10cm2 per vial) were cryopreserved in CryoStor CS10 medium (0.5 ml/vial; Stem Cell Technologies).

Differentiation of endothelial cells

hiPSC differentiation to ECs was performed as described previously (Orlova et al., 2014). Briefly, hiPSCs were maintained in mTeSR-E8 medium. For mesoderm induction (day 0-3), mTeSR-E8 medium was replaced with B(P)EL medium supplemented with 8 µM CHIR99021 (Tocris Bioscience, 4423). Cells were refreshed with vascular specification medium comprised of VEGF (50 ng/ml) and 10 µM SB431542 (Tocris Bioscience, 1614) in B(P)EL at day 3, day 6, and day 9. hiPSC-ECs were isolated on day 10 using CD31-Dynabeads™ (Thermo Fisher Scientific), as previously described (Orlova et al., 2014). hiPSC-ECs were expanded in complete EC growth medium (EC-CGM) comprised of Human Endothelial-serum free medium (EC-SFM) with 1% Human platelet poor serum (P2918, Sigma), VEGF (30 ng/ml) and bFGF (20 ng/ml), as described previously with minor modifications (Orlova et al., 2014). hiPSC-ECs were expanded for additional 3-4 days post-isolation and cryopreserved using cryopreservation medium consists of 50% fetal bovine serum,40% EGM2 and 10% dimethyl sulfoxide at passage number 1 (P1) (StemCell Technologies, 07930).

3D cardiac microtissue formation

MTs were formed as described previously (Giacomelli et al., 2020). Prior to MT formation, hiPSC-ECs and hiPSC-CFs were prepared as follows: 3-4 days before MT formation, a vial of cryopreserved hiPSC-ECs and a vial of cryopreserved hiPSC-CFs were thawed and cultured either in EC-CGM on plates coated with gelatin (hiPSC-ECs), or in FGM3 on uncoated plates (hiPSC-CFs). On the day of MT formation (day 0), hiPSC-ECs and hiPSC-CFs were detached using TrypLE 1X for 2-3 mins at RT (EC) and 5 mins at 37 °C, 5 % CO2 (CF), centrifuged for 3 min at 1100 rpm, resuspended in B(P)EL medium and counted. hiPSC-CMs at day 14-21 that showed > 80 % purity, measured as the percentage of troponin positive cells by FACS, were dissociated using the TryplE 5X for 10 mins at 37 °C, 5 % CO2, resuspended in B(P)EL medium and counted. Cell suspensions were combined to a total of 5000 cells (70 % CM, 15 % EC and 15 % CF) per 50 µl B(P)EL medium supplemented with VEGF (50 ng/ml) and FGF2 (5 ng/ml). For all MTs, cell suspensions were seeded on V-bottomed 96-well microplates (Greiner bio-one) and centrifuged for 10 min at 1100 rpm. MTs were incubated at 37 °C, 5 % CO2 for 12 days with media refreshed every 3-4 days.

Cell Preparation before on chip culture

Human brain vascular pericytes (HBVPs) were purchased from ScienceCell. HBVPs were cultured in Pericyte Medium (ScienceCell, 1201) supplemented with Pericyte Growth Supplement (ScienceCell, 1252) and 2 % FBS. Cells were cryopreserved at passage number 3 (P3) using serum-free cryopreservation medium (CryoStor™CS10) (StemCell Technologies, 07930). hiPSC-ECs (P1) were thawed and cultured on gelatin-coated plates in EC-CGM, 4 days prior to VoC seeding. HBVPs (P3) were thawed and cultured in Pericyte Medium (ScienceCell, 1201) supplemented with Pericyte Growth Supplement (ScienceCell, 1252) and 2 % FBS.

L-NAME and IL-1β stimulation

On the day of L-NAME stimulation chips were refreshed with the medium supplemented with either VEGF (50 ng/mL) and distilled water (Vehicle) or VEGF (50 ng/mL) and L-NAME (1 mM, N5751, Sigma) for one or six hours. Chips were incubated at 37 °C, 5% CO2 and contraction analyses were performed after one hour or six hours.

On the day of IL-1β stimulation chips were refreshed with the medium supplemented with either VEGF (50 ng/mL) and PBS (Vehicle) or VEGF (50 ng/mL) and IL-1β (10 ng/ml, N5751, Sigma) for twelve hours. Chips were incubated at 37 °C, 5% CO2 and contraction analyses were performed after twelve hours.

Multiplex Cytokine Analysis

The medium from multiple chips were collected after twelve hours stimulation with IL-1β (10 ng/ml) or vehicle, and kept at -80 ºC. On the day of the multiplex cytokine bead assay, collected medium was thawed two hours prior to the experiment. Concentration of cytokines (CXCL10, IL-1β, TNFα, CCL2, IL-6, IL-10, IFNϒ, TGFβ1 free active and IL-8) was measured using a LEGENDplex Human Essential Immune Response Panel kit (13-plex) (BioLegend, cat no: 740930) according to the manufacturer's instructions. Undiluted or eight times diluted samples were run on Cytek 3-Laser Aurora spectral flow cytometer (Cytek Biosciences, USA).

Imaging of vascularized 3D cardiac microtissues on chip

Whole channel images were captured daily with EVOSM7000 using 10x objectives. For automated imaging of whole channels and stitching, a customized plate layout was used. VMToCs and MToCs were fixed for 30 mins at 4 °C with 4% paraformaldehyde, washed 3 times in PBS (Calcium, Magnesium) and stored at 4 °C until processing. Confocal images were captured to create 3D stack using Andor Dragonfly spinning disk confocal microscope using a 20x or 40x objective. Image processing was done using Imaris 9.5 software (Bitplane, Oxford Instruments).

Characterization of vascular networks

Fields of views (FOV) from the full channels (acquired using EVOS) were arranged in size of 480X1200 pixels around the MTs. For VoC 75 FOVs and for VMToC 87 FOVs were captured from three independent experiments with at least six microfluidic channels each. These FOVs (Figure 1E-H), whole microfluidic channels (Figure S2D), and 10x images of MTs from channels (Figure S2E) were then quantified using pipelines developed on the free open source CellProfiler software (https://cellprofiler.org/) (Carpenter et al., 2006; Vila Cuenca et al., 2021). Briefly, for vessel density, pre-processing steps were applied to all images to enhance image features and a gaussian filter to reduce unspecific object identification. Two filter steps were applied to images of vascular network to

reduce non-specific segmentation from cell junctions and a minimum cross-entropy thresholding method was used to produce a binarized image. The binarized images from the CellProfiler output were then analyzed using the freely available ImageJ software with a plugin (https://imagej.nih.gov/ij/ ,https://imagej.net/DiameterJ) (Hotaling et al., 2015).

Contraction analysis

For pacing experiments, electrodes were made in-house to fit to the gel channels of chips and used to pace MTs. MTs were stimulated at 0.8 and 1 Hz, with 15V/cm strength and 3 ms long stimulation pulse. Movies of spontaneous beating or paced MTs from VMToC and MToC conditions were acquired for at least 10 s at 37 °C either with a ThorLabs DCC3240M camera at 100 frames/s and a 10x objective phase contrast objective (Leica Inverted microscope IBDE), using the ThorLabs uc480 software (v 4.20), or with a Leica Microsystems LAS AF6000 microscope at 37 °C and 5 % CO2. On the day of L-NAME or IL-1β stimulation, VMToC and MToC conditions were recorded for their baseline spontaneous beating prior to drug administration. Chips were recorded after both one hour incubation and six hours incubation period with vehicle and L-NAME or twelve hours incubation period with IL-1β. Contraction data were obtained by analyzing movies with the MUSCLEMOTION ImageJ macro (ImageJ v. 2.0.0-rc-49) as described previously (Sala et al., 2018).

Sarcomere analysis

Confocal images of VMToC and MToC conditions were acquired using a DragonFly spinning disk (Andor) microscope with 40x objective. In each MT, one plane from each FOVs were chosen to quantify and converted to 8-bit images. For MToC, 88 FOVs were selected in 11 MTs and for VMToC 52 FOVs were selected in 8 MTs. Sarcomere quantification data was obtained by analyzing these 8-bit images with SotaTool, as described previously (Stein et al., 2022).

Fluorescent beads perfusion assay

Perfusion assessment of VMToC was done as described previously (Vila Cuenca et al., 2021). In brief, 405-beads (1 drop in 10 or 20 ml EGM-2, Fluoro-Max Dyed Blue Aqueous Fluorescent Particles, B0200, ThermoFisher Scientific) were added to the right medium port (100 µl) and to all other media ports (50 µl). Then, confocal image stacks and videos of the spontaneous move of the beads following the endothelial networks were acquired using DragonFly spinning disk (Andor) microscope with 20x and 40x magnification objective. Tracking of the beads was done using 3D rendering and cell tracking function in Imaris 9.5 software (Bitplane, Oxford Instruments).

Supplemental References

Berg, C.W. van den, Elliott, D.A., Braam, S.R., Mummery, C.L., and Davis, R.P. (2016). Differentiation of Human Pluripotent Stem Cells to Cardiomyocytes Under Defined Conditions. Methods Mol. Biol. *1353*, 163–180.

Carpenter, A.E., Jones, T.R., Lamprecht, M.R., Clarke, C., Kang, I.H., Friman, O., Guertin, D.A., Chang, J.H., Lindquist, R.A., Moffat, J., et al. (2006). CellProfiler: image analysis software for identifying and quantifying cell phenotypes. Genome Biol. *7*, R100.

Giacomelli, E., Bellin, M., Orlova, V. V, and Mummery, C.L. (2017). Co-Differentiation of Human Pluripotent Stem Cells-Derived Cardiomyocytes and Endothelial Cells from Cardiac Mesoderm Provides a Three-Dimensional Model of Cardiac Microtissue. Curr. Protoc. Hum. Genet. *95*, 21.9.1-21.9.22.

Giacomelli, E., Meraviglia, V., Campostrini, G., Cochrane, A., Cao, X., van Helden, R.W.J., Krotenberg Garcia, A., Mircea, M., Kostidis, S., Davis, R.P., et al. (2020). Human-iPSC-Derived Cardiac Stromal Cells Enhance Maturation in 3D Cardiac Microtissues and Reveal Non-cardiomyocyte Contributions to Heart Disease. Cell Stem Cell *26*, 862-879.e11.

Guadix, J.A., Orlova, V. V, Giacomelli, E., Bellin, M., Ribeiro, M.C., Mummery, C.L., Pérez-Pomares, J.M., and Passier, R. (2017). Human Pluripotent Stem Cell Differentiation into Functional Epicardial Progenitor Cells. STEMCR *9*, 1754–1764.

Hotaling, N.A., Bharti, K., Kriel, H., and Simon, C.G. (2015). DiameterJ: A validated open source nanofiber diameter measurement tool. Biomaterials *61,* 327-338.

Orlova, V. V., Van Den Hil, F.E., Petrus-Reurer, S., Drabsch, Y., Ten Dijke, P., and Mummery, C.L. (2014). Generation, expansion and functional analysis of endothelial cells and pericytes derived from human pluripotent stem cells. Nat. Protoc. *9,* 1514-1531

Rostovskaya, M., Fu, J., Obst, M., Baer, I., Weidlich, S., Wang, H., Smith, A.J.H., Anastassiadis, K., and Francis Stewart, A. (2012). Transposon-mediated BAC transgenesis in human ES cells. Nucleic Acids Res. *40,* e150.

Sala, L., van Meer, B.J., Tertoolen, L.G.J., Bakkers, J., Bellin, M., Davis, R.P., Denning, C., Dieben, M.A.E., Eschenhagen, T., Giacomelli, E., et al. (2018). Musclemotion: A versatile open software tool to quantify cardiomyocyte and cardiac muscle contraction in vitro and in vivo. Circ. Res. *122,* e5-e16.

Stein, J.M., Arslan, U., Franken, M., De Greef, J.C., Harding, S.E., Mohammadi, N., Orlova, V. V, Bellin, M., Mummery, C.L., and Van Meer, B.J. (2022). Software Tool for Automatic Quantification of Sarcomere Length and Organization in Fixed and Live 2D and 3D Muscle Cell Cultures In Vitro. Curr. Protoc. Hum. Genet. *2,* e462.

Vila Cuenca, M., Cochrane, A., van den Hil, F.E., de Vries, A.A.F., Lesnik Oberstein, S.A.J., Mummery, C.L., and Orlova, V. V. (2021). Engineered 3D vessel-on-chip using hiPSC-derived endothelial- and vascular smooth muscle cells. Stem Cell Reports *16,* 2159-2168.

Zhang, M., D'Aniello, C., Verkerk, A.O., Wrobel, E., Frank, S., Ward-Van Oostwaard, D., Piccini, I., Freund, C., Rao, J., Seebohm, G., et al. (2014). Recessive cardiac phenotypes in induced pluripotent stem cell models of Jervell and Lange-Nielsen syndrome: Disease mechanisms and pharmacological rescue. Proc. Natl. Acad. Sci. U. S. A. *111,* e5383-e5392.
